# Adsorption of Sulfur Dioxide in Cu(II)-Carboxylate Framework Materials: The Role of Ligand Functionalization and Open Metal Sites

**DOI:** 10.1021/jacs.2c03280

**Published:** 2022-07-18

**Authors:** Weiyao Li, Jiangnan Li, Thien D. Duong, Sergei A. Sapchenko, Xue Han, Jack D. Humby, George F. S. Whitehead, Iñigo J. Victórica-Yrezábal, Ivan da Silva, Pascal Manuel, Mark D. Frogley, Gianfelice Cinque, Martin Schröder, Sihai Yang

**Affiliations:** † Department of Chemistry, University of Manchester, Manchester M13 9PL, U.K.; ‡ ISIS Facility, STFC Rutherford Appleton Laboratory, Chilton, Oxfordshire OX11 0QX, U.K.; § Diamond Light Source, Harwell Science and Innovation Campus, Oxfordshire OX11 0DE, U.K.

## Abstract

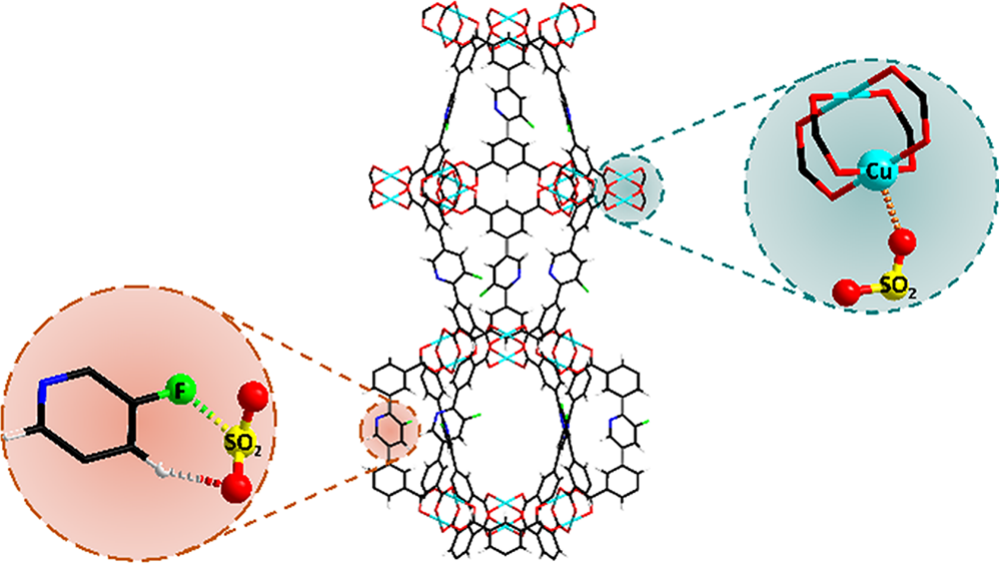

The development of efficient sorbent materials for sulfur dioxide (SO_2_) is of key industrial interest. However, due to the corrosive nature of SO_2_, conventional porous materials often exhibit poor reversibility and limited uptake toward SO_2_ sorption. Here, we report high adsorption of SO_2_ in a series of Cu(II)-carboxylate-based metal–organic framework materials. We describe the impact of ligand functionalization and open metal sites on the uptake and reversibility of SO_2_ adsorption. Specifically, MFM-101 and MFM-190(F) show fully reversible SO_2_ adsorption with remarkable capacities of 18.7 and 18.3 mmol g^–1^, respectively, at 298 K and 1 bar; the former represents the highest reversible uptake of SO_2_ under ambient conditions among all porous solids reported to date. *In situ* neutron powder diffraction and synchrotron infrared microspectroscopy enable the direct visualization of binding domains of adsorbed SO_2_ molecules as well as host–guest binding dynamics. We have found that the combination of open Cu(II) sites and ligand functionalization, together with the size and geometry of metal–ligand cages, plays an integral role in the enhancement of SO_2_ binding.

## Introduction

Fossil fuels will continue to dominate the energy landscape in the decades to come, leading to significant emissions of SO_2_.^1^ Air pollution by SO_2_ has detrimental effects on both human health and the environment,^2−5^ and SO_2_ in the atmosphere is thus a major source of pollution and is associated with global climate change.^3,4^ SO_2_ is also an important industrial feedstock primarily for the manufacture of sulfuric acid, which uses 98% of the total production of SO_2_.^6^ Although the state-of-the-art flue-gas desulfurization (FGD) technologies can remove up to 95% SO_2_, they generate a tremendous amount of solid waste, and residual SO_2_ can later poison CO_2_ scrubbers downstream of FGD processes.^7^ Regenerable methods can mitigate the production of waste by recycling the sorbent post SO_2_ adsorption, and the recovered SO_2_ can be used further for the synthesis of sulfuric acid. Sorbent materials with high SO_2_ capacity can be used as a safe host for the transport of SO_2_, eliminating the energy cost for its reduction to elemental sulfur followed by re-oxidation to SO_2_.^6^ Traditional porous materials including metal oxides,^8^ activated carbons,^9^ and zeolites^10^ have been tested for SO_2_ adsorption. However, these materials tend to demonstrate low SO_2_ capacities under ambient conditions (usually in the range of 1–5 mmol g^–1^) owing to their limited surface areas and often they undergo irreversible structural degradation upon the harsh conditions required to remove adsorbed or bound SO_2_.^11^

Metal–organic frameworks (MOFs) are promising sorbent materials owing to their exceptional surface area and tuneable pore environments.^12,13^ Functionalization of the organic linker and/or incorporation of coordinatively unsaturated metal sites can deliver targeted properties to the resultant MOFs, such as preferential adsorption of H_2_, CH_4_, CO_2_, and light hydrocarbons.^14−18^ The use of MOF materials as SO_2_ sorbents is currently a rapidly developing field of study.^19−32^ MOF-177 exhibits a record high SO_2_ uptake of 25.7 mmol g^–1^ at 298 K and 1 bar, but it shows irreversible structural degradation upon desorption.^22^ Considering that over 100,000 MOFs are reported to date,^33^ samples that are stable to repeated exposure to SO_2_ are still relatively rare, including MFM-300(In) (8.28 mmol g^–1^),^23^ MFM-300(Sc) (9.4 mmol g^–1^),^24^ DMOF (9.97 mmol g^–1^),^25^ NU-1000 (10.9 mmol g^–1^),^26^ SIFSIX-1-Cu (11.0 mmol g^–1^),^27^ NU-200 (11.7 mmol g^–1^),^28^ MFM-601 (12.3 mmol g^–1^),^29^ MFM-300(Sc)@EtOH (13.2 mmol g^–1^),^24^ MOF-808 (15.3 mmol g^–1^),^30^ MFM-170 (17.5 mmol g^–1^),^31^ and MIL-101(Cr)-4F(1%) (18.4 mmol g^–1^)^32^ (uptake given at 298 K and 1 bar of SO_2_). Systems incorporating open metal sites that are capable of capturing SO_2_ are extremely rare.^31^ Thus, the optimization of pore environment in terms of ligand functionalization, implementation of open metal sites, and control of pore geometry is an important approach to achieve reversible, high adsorption of SO_2_.

Here, we report a comprehensive investigation of adsorption of SO_2_ in a series of Cu(II)-carboxylate-based MOFs, namely, MFM-100, MFM-101, MFM-102, MFM-126, MFM-190(F), MFM-190(NO_2_), MFM-190(CH_3_), and MFM-190(H) (published previously as ZJU-5), showing metal–ligand cages of different sizes and showing combinations of open Cu(II) sites and functional groups within the cages. MFM-126 has a (3,6)-connected framework with an *eea* topology with [Cu_2_(OOCR)_4_] paddlewheels bound to carboxylate and pyrimidyl groups of the linker in the equatorial and axial positions, respectively, leaving no open Cu(II) sites. The other materials in this series crystallize in *nbo* topology with [Cu_2_(OOCR)_4_] paddlewheels bound to carboxylate donors of the linker and water molecules at the equatorial and axial positions, respectively. Open Cu(II) sites can be generated by removal of the terminally bound water molecules by heating under vacuum. MFM-101 and MFM-190(F), the latter with a fluoro-functionalization, show fully reversible adsorption of SO_2_ of 18.7 and 18.3 mmol g^–1^ at 298 K and 1 bar, respectively; the former represents the highest reversible uptake of SO_2_ in porous solids. These two materials also show high stability toward cyclic adsorption and desorption of SO_2_, retaining full crystallinity and uptake capacity over multiple cycles. The other systems show a decrease in uptake, porosity, or crystallinity upon repeated cycles of adsorption–desorption. The host–guest binding interaction and locations of adsorbed SO_2_ molecules in MFM-190(F) and MFM-126 have been visualized by *in situ* neutron powder diffraction (NPD), inelastic neutron scattering (INS), and synchrotron infrared microspectroscopy studies. These reveal that a combination of open Cu(II) sites and ligand functionalization, along with the size and geometry of metal–ligand cages, results in the exceptional adsorption of SO_2_ in MFM-190(F).

## Results and Discussion

### Synthesis and Structural Analysis

**Figure 1:**
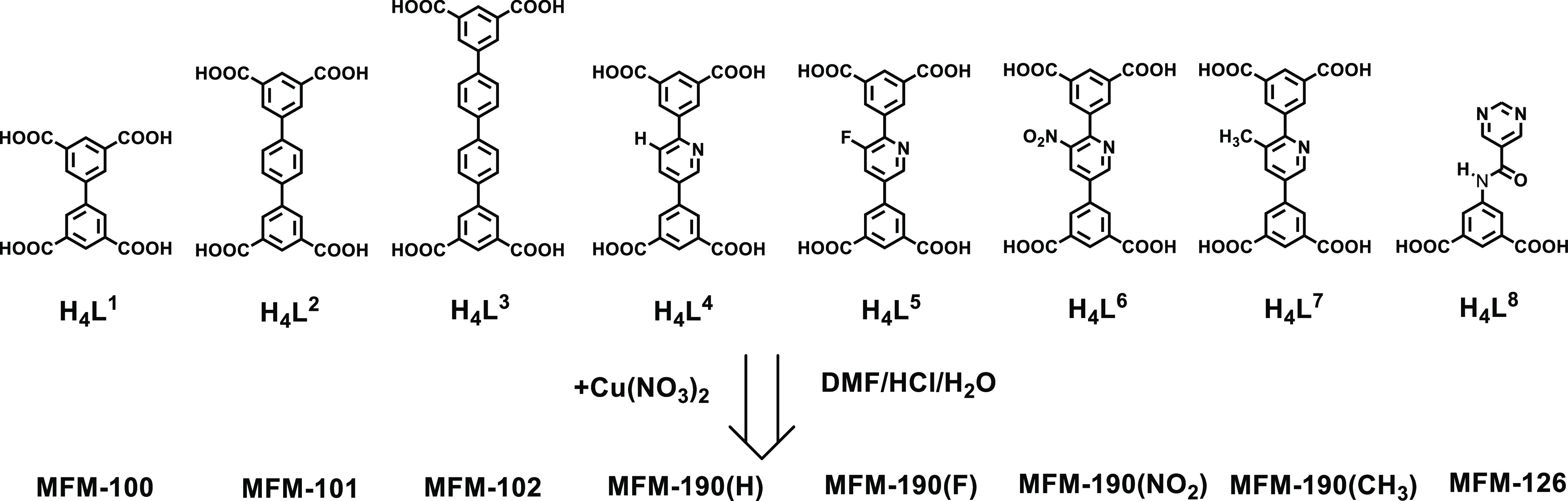
Structures of H_4_L^1^–H_4_L^8^ and nomenclature for their corresponding Cu(II)-based MOFs.

**Figure 2:**
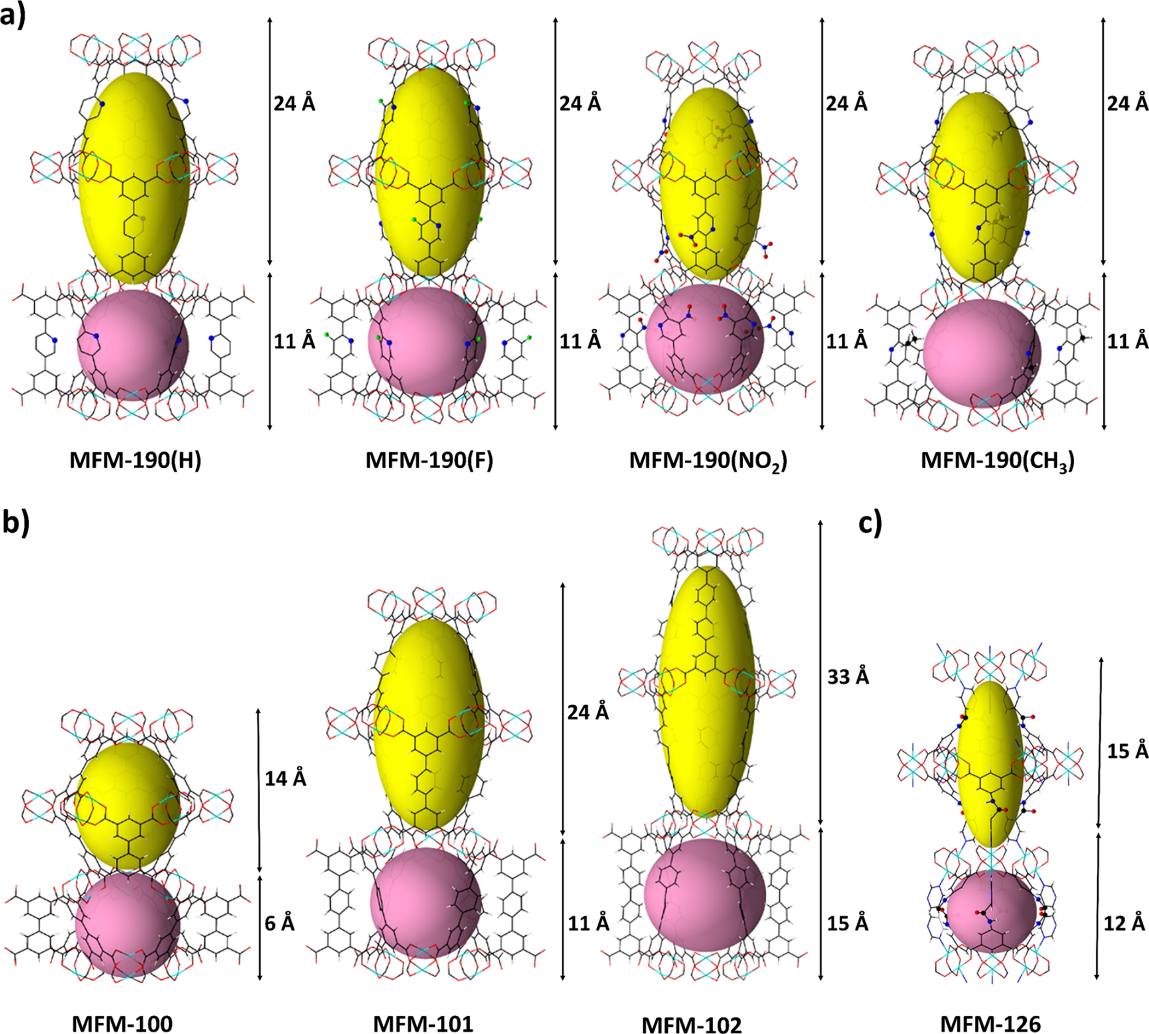
Views of the metal–organic cages in the MOFs in this study. Views of (a) MFM-190(H), MFM-190(F), MFM-190(NO_2_), and MFM-190(CH_3_) and (b) MFM-100, MFM-101, and MFM-102, which adopt the *nbo* network topology with open metal sites. (c) View of MFM-126, which adopts the *eea* network topology with no open metal sites. Cu, cyan; C, black; O, red; H, white; N, blue; F, green. Void spaces are highlighted in yellow (cage A) and pink (cage B); the given lengths refer to the sizes of the cages.

Three new MOFs, namely, MFM-190(F), MFM-190(NO_2_), and MFM-190(CH_3_), along with previously reported^34−36^ MFM-100, MFM-101, MFM-102, MFM-190(H), and MFM-126, were synthesized *via* solvothermal reactions of carboxylate ligands and Cu(NO_3_)_2_ in dimethylformamide (DMF) (Figure 1). Powder X-ray diffraction (PXRD) confirmed the phase purity of all bulk samples (Figures S1–S4). The PXRD patterns confirm that MFM-190(F), MFM-190(NO_2_), and MFM-190(CH_3_) are iso-structural to MFM-190(H) and MFM-101 (Figures S5 and S6). Single-crystal X-ray diffraction shows that MFM-190(F), MFM-190(NO_2_), and MFM-190(CH_3_) crystallize in the trigonal space group *R*-3*m* and shows *nbo* network topology. The asymmetric unit contains one Cu(II) ion, one quarter of a tetracarboxylate linker, and one terminal water molecule, and the structure is formed by bridging [Cu_2_(OOCR)_4_] paddlewheels incorporating di-isophthalate linkers. The resulting framework is composed of two different types of cages in a 1:1 ratio. A cylindrical cage A is constructed by 12 [Cu_2_(OOCR)_4_] paddlewheels and six linkers with a length of *ca.* 24 Å and width of 18 Å at the equatorial ring. A spherical cage B is made up of six [Cu_2_(OOCR)_4_] paddlewheels and six linkers with a diameter of *ca.* 11 Å (Figure 2a). These cages are decorated with −H, −F, −NO_2_, and −CH_3_ groups in MFM-190(H), MFM-190(F), MFM-190(NO_2_), and MFM-190(CH_3_), respectively, as well as pyridyl N centers. These four MOFs have similar thermal stability up to ∼300 °C (Figure S7)^34^ and are iso-reticular to MFM-100, MFM-101, and MFM-102,^35^ which also adopt an *nbo* network topology. The linkers of MFM-100, MFM-101, and MFM-102 increase in length along the series with the cage A increasing in length from 14 Å in MFM-100 to *ca.* 33 Å in MFM-102 and cage B increasing in length from 6 Å in MFM-100 to *ca.* 15 Å in MFM-102 as the linker elongates (Figure 2b). MFM-126 crystallizes in the trigonal space group R-3 and adopts a (3,6)-connected framework with an *eea* network topology. Similar to the *nbo*-type MOFs, MFM-126 is made up of two types of cages, a cylindrical cage A made up of 12 [Cu_2_(OOCR)_4_] paddlewheels and six linkers with a length of *ca.* 15 Å and a spherical cage B made up of six [Cu_2_(OOCR)_4_] paddlewheels and six linkers with a diameter of *ca.* 12 Å. However, the axial positions of the [Cu_2_(OOCR)_4_] paddlewheels are capped by pyrimidyl nitrogen centers of the linker, and therefore, no open Cu(II) metal sites are generated upon activation of MFM-126 (Figure 2c).

### Analysis of Gas Adsorption Isotherms and Dynamic Separation of SO_2_

The BET surface areas of desolvated MFM-100, MFM-101, MFM-102, MFM-126, MFM-190(F), MFM-190(NO_2_), MFM-190(CH_3_), and MFM-190(H) were determined by N_2_ adsorption isotherms at 77 K to be 1445, 2300, 2873, 965, 2538, 2304, 2550, and 2373 m^2^ g^–1^, respectively. The increase in BET surface area from MFM-100 to MFM-102 is due to the expansion of metal–organic cages as a result of the elongation of the ligands, while ligand functionalization appears to have a limited impact on the BET surface areas of MFM-190(F), MFM-190(NO_2_), MFM-190(CH_3_), and MFM-190(H) (Figure S8).

**Figure 3:**
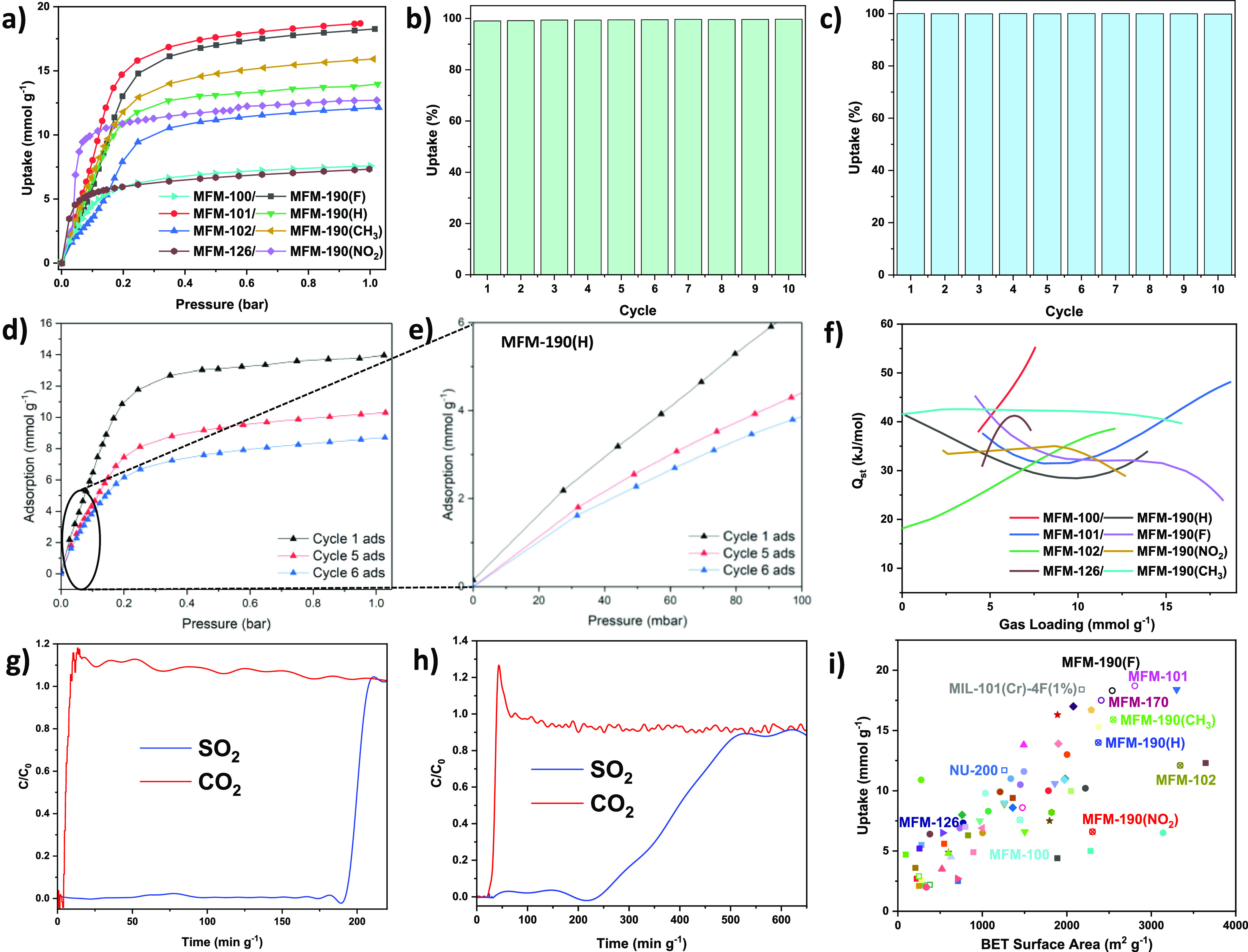
(a) SO_2_ adsorption isotherms at 298 K and 1 bar for all materials in this study (desorption isotherms are omitted for clarity and shown in the SI). Cycling of SO_2_ between 0 and 500 mbar at 298 K in (b) MFM-190(F) and (c) MFM-101. (d) Comparison of adsorption of SO_2_ at 298 K for MFM-190(H) over different cycles at 1 bar. (e) View of adsorption of SO_2_ at 298 K for MFM-190(H) over different cycles at 100 mbar. (f) Values for *Q*_st_ for SO_2_ in all materials in this study. Breakthrough plots for mixtures of SO_2_/CO_2_ (SO_2_/CO_2_: 2500 ppm/15%; total flow rate: 30 mL min^–1^) over a fixed-bed packed at 298 K with (g) MFM-190(F) and (h) MFM-101. (i) Plot of the BET surface area against the SO_2_ adsorption at 1 bar and 298 K for selected MOF materials that show reversible adsorption of SO_2_ (full details are given in Table S3).

The gravimetric adsorption isotherms of SO_2_ have been recorded for all eight MOFs at 273 and 298 K and from 0 to 1 bar (Figures S11–S18). MFM-101 shows a SO_2_ uptake of 20.8 mmol g^–1^ (or 1.33 g g^–1^) at 273 K and 1.0 bar, exceeding those reported for all leading sorbent materials for SO_2_ under the same conditions, such as MFM-170 (19.4 mmol g^–1^),^31^ MFM-601 (16.9 mmol g^–1^),^29^ and MFM-202a (12.8 mmol g^–1^).^37^ At 298 K and 1 bar, MFM-100, MFM-101, MFM-102, MFM-126, MFM-190(F), MFM-190(NO_2_), MFM-190(CH_3_), and MFM-190(H) show SO_2_ uptakes of 7.6, 18.7, 12.1, 7.33, 18.3, 12.7, 15.9, and 14.0 mmol g^–1^, respectively (Figure 3a). Multiple cycles of adsorption–desorption have been tested for all samples at 298 K to identify the reversibility and stability of adsorption. MFM-190(H) displays a loss of 38% in the uptake capacity of SO_2_ at 1 bar over six cycles (Figure 3d,e), which is accompanied by a decrease in the BET surface area from 2373 to 1959 m^2^ g^–1^ (Figure S9). The relatively small decrease in porosity (17%) compared with that in SO_2_ uptake (38%) suggests a loss of active sites and porosity due to pore collapse. Analysis of the pore size distribution (PSD) suggests that the pores of pristine MFM-190(H) are centered at around 5 Å and 7–9 Å (Figure S9b), while post-SO_2_ exposure, multiple irregular pores are generated with diverse diameters between 15–18 Å and 25–35 Å.

Incorporation of −F, −NO_2_, and −CH_3_ functionalization has varying impacts on SO_2_ adsorption with uptakes of 18.3, 12.7, and 15.9 mmol g^–1^ recorded at 298 K and 1 bar in MFM-190(F), MFM-190(NO_2_), and MFM-190(CH_3_), respectively, compared with the 14.0 mmol g^–1^ for the parent MFM-190(H) (Figure 3a). While showing steep adsorption at a low pressure (10.3 mmol g^–1^ at 0.1 bar), MFM-190(NO_2_) suffers severe framework collapse upon desorption; the sample post several cycles of adsorption–desorption of SO_2_ shows a marked reduction of the BET surface area from 2304 to 1172 m^2^ g^–1^ (Figure S10). In contrast, MFM-190(F) and MFM-190(CH_3_) both show consistent adsorption capacity of SO_2_ over 10 cycles (Figure 3b and Figure S17). However, the comparison of PXRD gives distinct results: while MFM-190(CH_3_) largely loses its crystallinity post-SO_2_ exposure, MFM-190(F) fully retains its crystallinity (Figure S19f,h). Thus, the introduction of −F groups to the linker has greatly enhanced the framework stability of MFM-190(F).

MFM-100, MFM-101, and MFM-102 exhibit excess SO_2_ adsorption capacities of 7.6, 18.7, and 12.1 mmol g^–1^ at 298 K and 1 bar (Figure 3a). A 3.7% loss in uptake capacity over 10 cycles of SO_2_ adsorption at 298 K is observed in MFM-100 (Figure S11b) with the post-SO_2_ exposure sample showing broadening and loss of Bragg peaks by PXRD due to reduction in crystallinity and structural order of the sample (Figure S19a). However, MFM-101 shows fully reversible SO_2_ uptake over 10 cycles without any loss in total uptake capacity (Figure 3c). Indeed, comparison of the PXRD pattern for the as-synthesized and post-SO_2_ cycling sample of MFM-101 confirmed little change in crystallinity (Figure S19b). Interestingly, although MFM-102 only shows a 5.6% loss in uptake capacity over 10 cycles of SO_2_ adsorption at 298 K (Figure S13b), comparison of the PXRD patterns of the fresh and post-SO_2_ exposure MFM-102 samples shows that there is a significant loss in long-range structural order in the latter (Figure S19c).

MFM-126 displays an excess SO_2_ adsorption capacity of 7.3 mmol g^–1^ at 298 K and 1 bar (Figure 3a) and shows stable performance over 10 cycles of adsorption–desorption of SO_2_ with full reversibility (Figure S14b). This is also evidenced by the PXRD pattern of the post-SO_2_ sample of MFM-126, which fully retains its crystallinity (Figure S19d). However, compared with other MOFs, MFM-126 shows a much lower uptake capacity of SO_2_, reflecting potentially the lack of open metal sites.

The ability to capture SO_2_ at low concentrations by MFM-190(F) and MFM-101 was tested by dynamic breakthrough experiments with SO_2_-containing gas mixtures at 298 K and 1 bar. Both MFM-190(F) and MFM-101 display excellent dynamic adsorption of SO_2_ at a low concentration (2500 ppm SO_2_) (Figures S20 and S21). In addition, MFM-190(F) and MFM-101 successfully capture SO_2_ from a mixture of 15% CO_2_ and 2500 ppm SO_2_ with the breakthrough time for SO_2_ of 190 and 230 min g^–1^, respectively (Figure 3g,h). The dynamic selectivities of SO_2_/CO_2_ are estimated to be 5.2 and 2.5 for MFM-190(F) and MFM-101, respectively (Table S3). This result further confirms the ability of MFM-190(F) and MFM-101 to selectively capture SO_2_ with an efficiency down to <0.1 ppm from 2500 ppm in a single adsorption cycle under dry conditions. Furthermore, the excellent stability of MFM-190(F) has been demonstrated by three cycles of breakthrough separations of SO_2_/N_2_ (2500 ppm SO_2_) (Figure S20).

The performance of the state-of-the-art porous materials for SO_2_ adsorption under ambient conditions is summarized in Table S3 and Figure 3i. Of the MOFs with *nbo* topology, MFM-101 and MFM-190(F) exhibit the most promising stability and uptake capacity, comparable to those of MIL-101(Cr)-4F(1%), which is the previous record holder for SO_2_ adsorption at 298 K and 1 bar for a porous material that is stable to repeated SO_2_ cycling.^32^ Unlike MIL-101(Cr)-4F(1%), which does not show saturation at 1 bar, the isotherms for MFM-190(F) and MFM-101 both exhibit a reversible type I profile. MIL-101(Cr)-4F(1%) exhibits apparent hysteresis of up to 2 mmol g^–1^ during desorption at 298 K below 800 mbar, attributed to the relatively strong SO_2_-framework interaction, as evidenced by the calculated heat of adsorption (*Q*_st_) of 54.3 kJ mol^–1^.^32^ In MFM-190(F) and MFM-101, hysteresis during desorption is less dramatic and begins around 200 mbar. The value of *Q*_st_ for both MFM-190(F) and MFM-101 is around 35–40 kJ mol^–1^ (Figure 3f), notably weaker than that of MIL-101(Cr)-4F(1%), consistent with a narrower hysteresis and a more facile desorption with a lower energy penalty in the former pair of materials. The lack of functional groups in MFM-190(H) results in a lower value for *Q*_st_ of 29–41 kJ mol^–1^ for SO_2_ adsorption. With increasing loading of SO_2_, the MFM-190 materials display a decrease in *Q*_st_, indicating reduced host–guest interactions as the primary binding sites become occupied. MFM-100 and MFM-102 exhibit increases in *Q*_st_ upon SO_2_ loading, which is likely related perhaps to the partial structural collapse/changes of the framework. The value of *Q*_st_ for MFM-101 decreases at low loadings and then increases upon further uptake of SO_2_, consistent with the presence of strong guest–guest interactions in the pore at high surface coverage and the high uptakes and stability. Owing to the absence of open Cu(II) sites, the value of *Q*_st_ for MFM-126 increases steadily with SO_2_ loading, reflecting the presence of host–guest interactions throughout the adsorption process.

### Determination of Binding Domains for Adsorbed SO_2_

**Figure 4:**
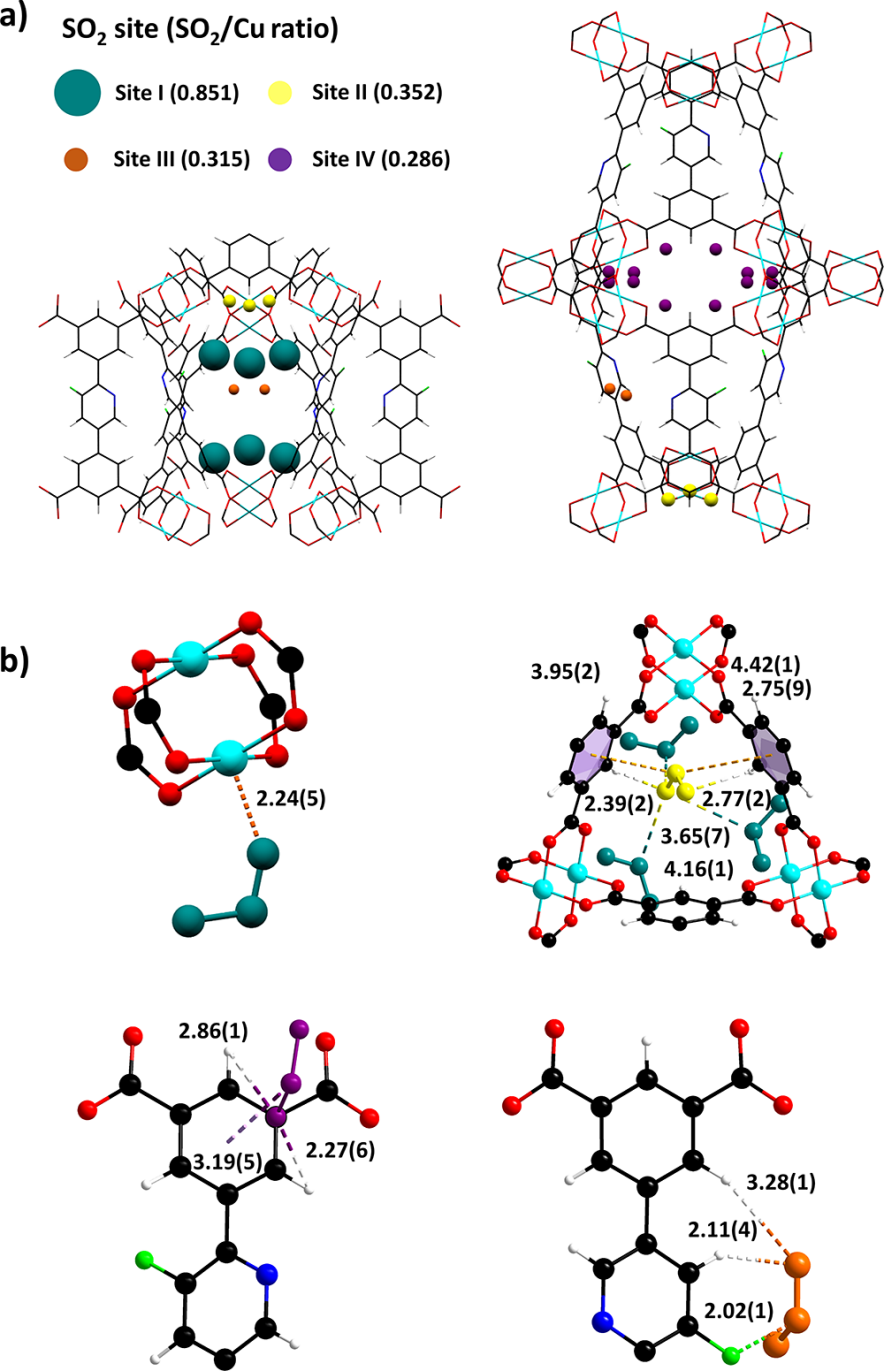
Views of the crystal structures of [Cu_2_C_21_H_12_FNO_10_·(SO_2_)_3.6_] derived from the NPD experiments. (a) Binding domains for SO_2_ in the cylindrical cage A and the spherical cage B of MFM-190(F). SO_2_ molecules are represented by a single sphere at the S positions and are scaled according to their occupancies (Site I: turquoise, Site II: yellow, Site III: pink, and Site IV: orange). (b) Views of binding sites for SO_2_ in MFM-190(F) (Site I: turquoise, Site II: yellow, Site III: magenta, and Site IV: orange). (The interatomic distances are in angstrom. Cu, cyan; C, black; O, red; H, white; N, blue; F, green).

**Figure 5:**
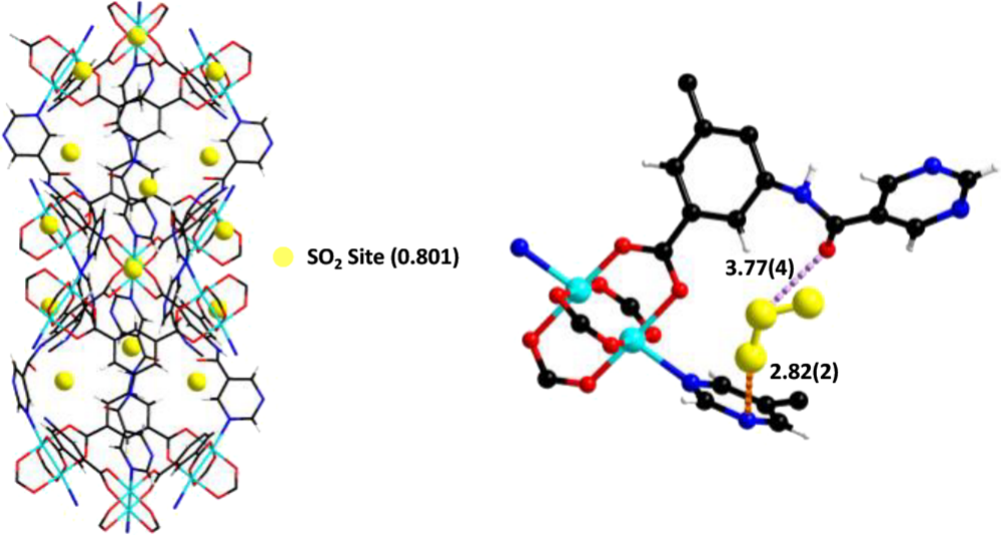
Views of the structures of [Cu_2_C_35_H_35_N_9_O_13_·(SO_2_)_1.6_] derived from the NPD experiment and of the primary binding site of SO_2_ in MFM-126. SO_2_ molecules are represented by a single sphere at the S positions (left) with a detailed view of the primary binding site (right). The interatomic distances are in angstrom. Cu, cyan; C, black; O, red; H, white; N, blue; SO_2_, yellow.

The adsorption domains of SO_2_ in MFM-190(F) and MFM-126 were determined by *in situ* NPD at 7 K in order to gain insights into the role of open Cu(II) sites and ligand functionalization. Structural analysis of desolvated materials confirms the complete removal of free solvents from the pores and of the coordinated water molecule on the Cu(II) sites of MFM-190, thus creating twelve and six open Cu(II) sites in cages A and B, respectively. Refinement of the NPD data of SO_2_-loaded MOFs revealed significant nuclear density within the pores. These were assigned as four (I–IV) distinct sites for SO_2_ in MFM-190(F) [Cu_2_(C_21_H_12_FNO_10_)·(SO_2_)_3.2_] (Figure 4) and one (I′) distinct site for SO_2_ in MFM-126 [Cu_2_(C_35_H_35_N_9_O_13_)·(SO_2_)_1.6_] (Figure 5).

In SO_2_-loaded MFM-190(F), the primary binding site, Site I (SO_2_/Cu = 0.785), is located in the spherical cage B, with SO_2_ interacting end-on to the open Cu(II) site [O_SO_2__–Cu = 2.24(5) Å, <S–O–Cu = 146°] (Figure 4). Site II (SO_2_/Cu = 0.296) sits approximately co-planar with the three [Cu_2_(OOCR)_4_] paddlewheels that define the boundary of the cylindrical and spherical cages, sandwiched between three phenyl rings that link the paddlewheels together. The SO_2_ molecule is located closer to one of the [Cu_2_(OOCR)_4_] paddlewheels than the other two, with a potential side-on interaction between the delocalized π systems of the two neighboring phenyl rings and S_SO_2__ [δ+, S_SO_2__···π_left_ = 3.95(2) Å, S_SO_2__···π_right_ = 4.42(1) Å]. Site II is further stabilized *via* two-fold hydrogen bonding between O_SO_2__ and the hydrogen atom on the phenyl ring [O_SO_2__···H–R = 2.39(2) and 2.77(2) Å, <Ȯ···H–C = 148 and 134°]. In addition, the dipole–dipole interactions [O_SO_2__···S_SO_2__ = 2.76(1), 3.65(7), and 4.16(1) Å] between SO_2_ molecules at Sites I and II further stabilize the packing of SO_2_. Interestingly, the primary and secondary sites for SO_2_ are opposite to those found in SO_2_-loaded MFM-170,^31^ where the open Cu(II) sites serve as secondary binding sites. One possible explanation for this is that half of the axial positions in MFM-170 are blocked by a pyridyl N-center from the linker, and some steric hindrance may be present around the open Cu(II) site. By contrast, all Cu(II) sites in MFM-190(F) can bind SO_2_, coupled with the presence of additional −F sites, contributing to the enhanced uptake of SO_2_.

Site III (SO_2_/Cu = 0.287) is found at the center of the cylindrical cage, located near the six terminal phenyl rings that connect the central three [Cu_2_(OOCR)_4_] paddlewheels. The SO_2_ molecule is offset to the phenyl ring and stabilized by dipole–dipole interactions between the delocalized π system and S_SO_2__ [O_SO_2__···π system = 3.19(5) Å] and electrostatic interactions between H from the phenyl rings and the O-center from the SO_2_ [R–H···O_SO_2__= 2.27(6) and 2.86(1) Å]. Site IV (SO_2_/Cu = 0.232) lies in the window between the cylindrical and spherical cages, sandwiched between two Lewis basic pyridyl rings. Interestingly, similar to that observed in SIFSIX,^28^ this binding site is stabilized by a side-on S^δ+^···F^δ−^ electrostatic interaction [S···F = 2.02(1) Å], coupled with two-fold hydrogen bonds between the O_SO_2__ and the hydrogen atom on the phenyl ring [O_SO_2__···H–R = 2.11(4) and 3.28(1) Å, <O···H–C = 143 and 139°]. This result confirms that beyond acting as an active site for SO_2_ binding, the primary role of the −F group is to increase the framework stability toward SO_2_ adsorption, consistent with the notable difference in the adsorption stability between MFM-190(F) and MFM-190(H).

A very small amount of SO_2_ was loaded into desolvated MFM-126 in order to probe the primary binding site in the absence of an open Cu(II) site. As a result, only one binding site (I′) for SO_2_ with a SO_2_/Cu ratio of 0.801 is observed and is located in the window between the larger cylindrical cage and the smaller spherical cage, similar to Site IV in MFM-190(F) (Figure 5). Site I′ in MFM-126 is stabilized *via* dipole–dipole interactions between S_SO_2__ and the oxygen of the amide group [S_SO_2__···O=C = 3.77(4) Å] as well as between O_SO_2__ and the nitrogen atom of the pyridine ring [O_SO_2__···N–R = 2.82(2) Å]. Both of these interactions appear to be weaker than those in Site IV of MFM-190(F), consistent with its low adsorption uptake.

Overall, the end-on binding interaction between SO_2_ and the open Cu(II) sites located at the axial positions of the [Cu_2_(OOCR)_4_] paddlewheels play a significant role in the high SO_2_ capacity of MFM-190(F), displaying the highest SO_2_/Cu ratio and the strongest host–guest interaction. In contrast, the primary binding site in MFM-126 is only weakly stabilized. Thus, the *in situ* NPD study has directly rationalized the adsorption performance of these materials.

### *In Situ* Spectroscopic Analysis of Host–Guest Binding Dynamics

**Figure 6:**
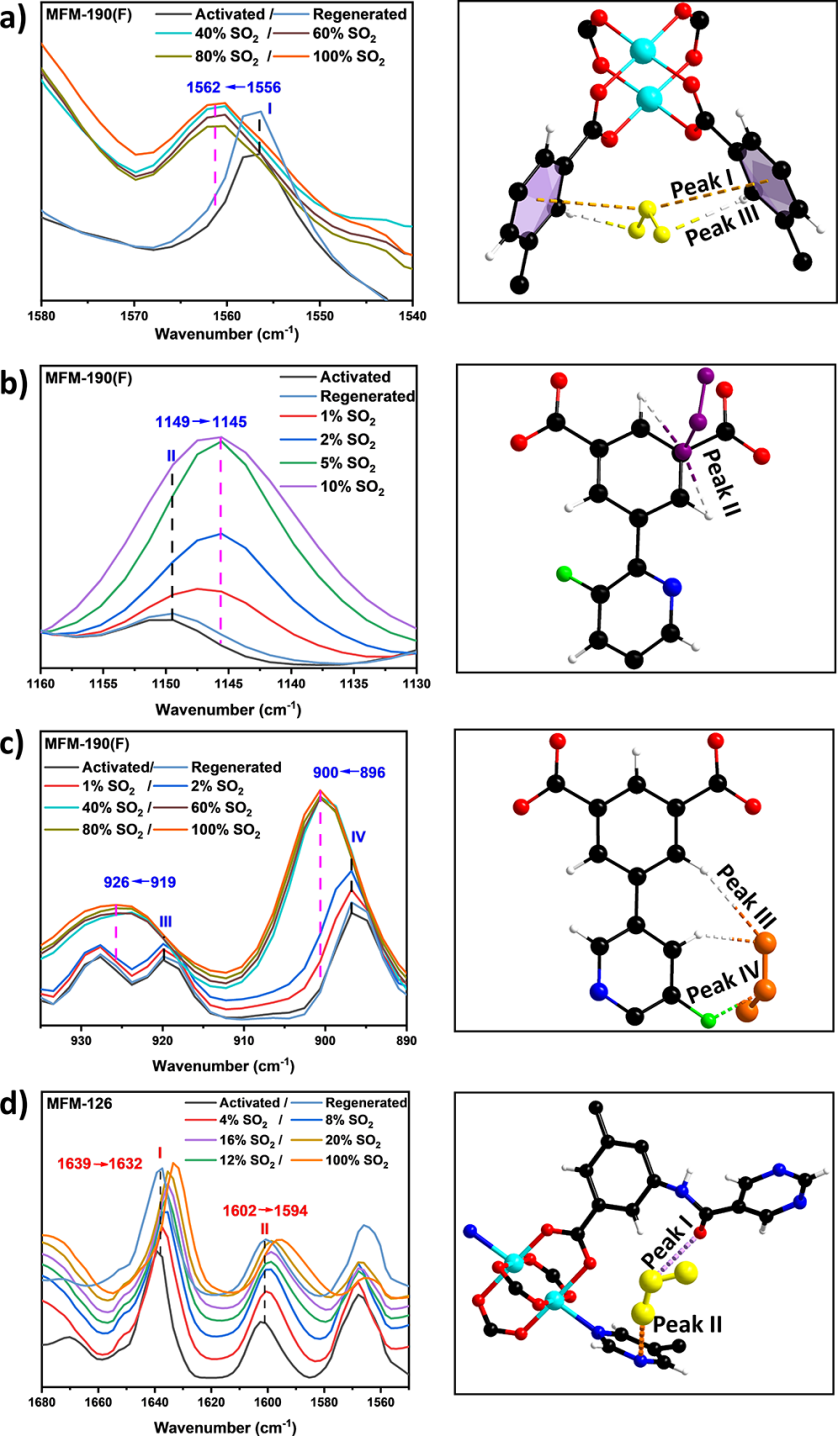
*In situ* synchrotron FTIR microspectra of selected MOFs as a function of SO_2_ adsorption (left) and views of the corresponding structural model (right). (a) Interaction with phenyl rings in MFM-190(F) at various loadings of SO_2_. (b) C–H in-plane mode in MFM-190(F) at various loadings of SO_2_. Data at high loading are omitted for clarity. (c) C–H out-of-plane mode and C–F stretching in MFM-190(F) at various loadings of SO_2_. (d) Stretching modes of C=O and C=N groups in MFM-126 at various loadings of SO_2_.

The binding dynamics of MFM-190(F), MFM-126, MFM-190(H), and MFM-101 upon adsorption of SO_2_ have been analyzed using *in situ* synchrotron infrared microspectroscopy (Figure 6). Desolvated MFM-190(F) shows a number of characteristic peaks at 1556, 1149, 919, and 896 cm^–1^ (denoted as I, II, III, and IV) (Figure 6a). Peak I is assigned to the distortion of the phenyl ring and Peaks II and III to the in-plane and out-of-plane bending modes of the aromatic C–H groups, respectively.^38^ Peak IV is assigned to the C–F stretching.^39^ On dosing with SO_2_ (0–1 bar), a blue shift of 6 cm^–1^ is observed for Peak I to 1562 cm^–1^, indicating a stiffening effect of the π system upon binding of SO_2_ molecules to the phenyl ring (Figure 6a). The red shift (Δ = 4 cm^–1^) of Peak II reflects the presence of −CH^δ+^···^δ−^OSO supramolecular contacts (Figure 6b). Furthermore, blue shifts of Peaks III and IV to 926 and 900 cm^–1^ (Δ = 7 and 4 cm^–1^, respectively) are observed, consistent with the presence of hydrogen bonds and dipole–dipole interaction as observed in crystallographic studies (Figure 6c).

Two characteristic peaks at 1639 and 1602 cm^–1^ are observed for desolvated MFM-126 (Figure 6d), which are assigned to the stretching modes of the C=O group and pyrimidine ring, respectively.^38^ Red shifts to 1632 and 1594 cm^–1^ (Δ = 7 and 8 cm^–1^, respectively) upon binding of SO_2_ suggest dipole–dipole interactions with C=O and C=N bonds, again consistent with the crystallographic model.

Desolvated MFM-190(H) (Figures S28–S32) shows a number of characteristic peaks at 1712, 1218, 1027, 838, and 680 cm^–1^ (denoted as I, II, III, IV, and V). Peak I is assigned to the stretching of the C=O bond and Peaks II and III to the symmetric and asymmetric stretching modes of the C−O bond, respectively. Peaks IV and V are assigned to the in-plane and out-of-plane bending modes of the C–H group of the aromatic ring, respectively. Notable shifts are observed upon SO_2_ dosing for Peak I (from 1712 to 1697 cm^–1^), Peak II (from 1218 to 1228 cm^–1^), and Peak III (from 1027 to 1022 cm^–1^), indicating interactions between adsorbed SO_2_ molecules and the carboxylate group.^38,40^ The blue shifts at Peak IV (from 838 to 850 cm^–1^) and Peak IV (from 680 to 686 cm^–1^) suggest the presence of hydrogen bonds between the aromatic C–H groups and adsorbed SO_2_ molecules.

Upon adsorption of SO_2_ in MFM-101, four bands experience an obvious shift (Figures S33–S35). The red shift at Peak I from 1637 to 1631 cm^–1^ suggests distortion of the phenyl ring due upon binding of SO_2_.^38,40^ The shift of Peak II from 1299 to 1311 cm^–1^ is consistent with the formation of supramolecular interactions between SO_2_ and the C–O group. The blue shifts of Peak III (from 838 to 844 cm^–1^) and Peak IV (from 769 to 773 cm^–1^) confirm the formation of host–guest hydrogen bonds with aromatic C–H groups. All changes are reversible upon regeneration under a flow of dry N_2_, consistent with the excellent reversibility and stability of SO_2_ adsorption in MFM-101.

## Conclusions

Remarkable and reversible adsorption of SO_2_ has been achieved by MFM-101 and MFM-190(F), 18.7 and 18.3 mmol g^–1^ at 298 K and 1 bar, respectively. Importantly, MFM-101 shows the highest reversible SO_2_ adsorption in porous solids to date. Additionally, MFM-101 and MFM-190(F) both show excellent stability toward multiple cycles of adsorption–desorption of SO_2_. The introduction of a −F group in MFM-190(F) significantly improves the stability and efficacy of the resultant framework upon SO_2_ adsorption compared with the non-functionalized MFM-190(H) as well as delivering additional binding sites. Open Cu(II) sites in MFM-190(F) were identified as the strongest adsorption sites for SO_2_ by Rietveld refinements of the *in situ* NPD data. Another MOF with exceptional stability toward SO_2_ is MFM-126, which has no open metal sites, although this results in a much lower observed capacity toward SO_2_ (7.3 mmol g^–1^ at 298 K and 1 bar) compared with MFM-190(F) and MFM-101. The dynamics involved in the binding of SO_2_ in MFM-126, MFM-190(F), MFM-101, and MFM-190(H) have been investigated by *in situ* spectroscopic techniques, further confirming the formation of supramolecular interactions with SO_2_. Studies of MFM-100, MFM-101, and MFM-102 suggest that a simple increase in surface area is not necessarily linked to the enhanced adsorption of SO_2_, in sharp contrast to the observation of their performance in H_2_ adsorption.^35^ These studies suggest that the collective contributions from open metal sites, ligand functionalization, and pore geometry have resulted in high and reversible adsorption of SO_2_ in these Cu(II)-carboxylate-based MOF materials.

## Supporting Information Available

Detailed synthesis, characterization and analysis of the crystal structures of all MOFs, TGA, porosity and heat of adsorption measurements, breakthrough experiments, FT-IR microspectroscopy, and Rietveld refinements of neutron diffraction data (PDF)
