# Supplementary material for: Adsorption of Sulfur Dioxide in Cu(II)-Carboxylate Framework Materials: The Role of Ligand Functionalization and Open Metal Sites
Source: J Am Chem Soc. 2022 Jul 18;144(29):13196–204. doi: 10.1021/jacs.2c03280 (PMC9345647; doi:10.1021/jacs.2c03280)
Supplement: Supplementary file 1 — ja2c03280_si_001.pdf [file ja2c03280_si_001.pdf]

Supporting Information

Adsorption of sulphur dioxide in Cu(II)-carboxylate framework materials: the role of ligand functionalisation and open metal sites

Weiyao Li,[1#] Jiangnan Li,[1#] Thien D. Duong,[1#] Sergei A. Sapchenko,[1] Xue Han,[1] Jack D. Humby,[1] George F. S. Whitehead,[1] Iñigo J. Victórica-Yrezábal ,[1] Ivan da Silva,[2] Pascal Manuel,[2] Mark D. Frogley,[3] Gianfelice Cinque,[3] Martin Schröder[1]* and Sihai Yang[1]*

1. Department of Chemistry, University of Manchester, Manchester, M13 9PL (UK)

2. ISIS Facility, STFC Rutherford Appleton Laboratory, Chilton, Oxfordshire, OX11 0QX (UK)

3. Diamond Light Source, Harwell Science and Innovation Campus, Oxfordshire, OX11 0DE (UK)

# These authors contributed to this work equally.

Contents

Synthesis and characterisation	S3
Experimental methods	S3
Single crystal X-ray analysis	S9
Powder X-ray diffraction	S10
Thermo-gravimetric analysis	S13
Characterisation of porosity and gas isotherms	S14
PXRD of MOF before and after SO_2 uptake	S20
Dynamic breakthrough separation of SO_2 in MFM-190(F) and MFM-101	S21
Analysis of heat of adsorption	S23
In situ FT-IR microscopy	S23
Rietveld refinement of NPD data	S31
Comparison of solid porous materials as sorbents for SO_2	S33
References	S35

Synthesis and characterisation

Experimental methods

[3,5-Bis(ethoxycarbonyl)phenyl]boronic acid and [3,5-bis(methoxycarbonyl)phenyl]boronic acid were obtained through the esterification of 3,5-dicarboxybenzeneboronic acid. All other chemicals and reagents were purchased through Sigma Aldrich, Fischer Scientific or Fluorochem and used as received without further purification. ^{1}H NMR and ^{13}C NMR spectra were measured on a Bruker AV400 or AV500 spectrometer. High-resolution electrospray mass spectra were measured on a Bruker Micro-TOF spectrometer with samples dissolved in MeOH using both positive and negative mode from m/z. Elemental analysis of ligands was carried out on a CE-440 elemental analyser (EAI Company). TGA measurements were performed using a Perkin Elmer TGA 7 Gravimetric Analyser under a flow of N_2 (20 ml min^{-1}) at a heating rate of 5 °C min^{-1}.

Synthesis of tetraethyl 5, 5'-(3-fluoropyridine-2, 5-diyl)diisophthalate (3)

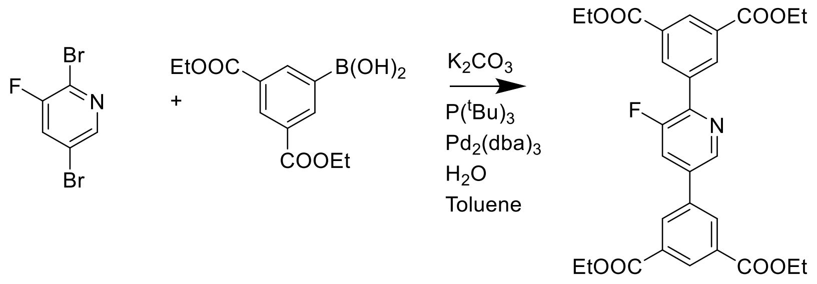

1 **2** **3**

A mixture of 2,5-dibromo-3-fluoropyridine (**1**) (1.27 g, 5.0 mmol), [3,5-bis(ethoxycarbonyl)phenyl]boronic acid (**2**) (3.19 g, 12.0 mmol), K_2CO_3 (3.36 g, 24 mmol), tri-*tert*-butylphosphine (3 mL), $Pd_2(dba)_3$ (0.89 g, 1.0 mmol), H_2O (85 mL) and toluene (350 mL) was heated at 80°C for 17 h, at 90°C for 30 min under N_2 and then cooled to room temperature. The reaction mixture was filtered, extracted with CH_2Cl_2 and the organic phase dried over $MgSO_4$. After filtration, the solvent was removed by evaporation and the residue purified by recrystallisation from CH_2Cl_2 and MeOH. The pure product **3** (2.14 g, 3.98 mmol, 79.6 %), was collected by filtration and dried in an oven, to afford a pale yellow solid. ^{1}H NMR (500 MHz, $CDCl_3$, ppm): δ 8.94 (t, J = 1.3 Hz, 2H), 8.91 (t, J = 1.6 Hz, 1H), 8.80 (t, J = 1.5 Hz, 1H), 8.78 (t, J = 1.5 Hz, 1H), 8.53 (d, J = 1.5 Hz, 2H), 7.86 (dd, J_1 = 11.7 Hz, J_2 = 1.9 Hz, 1H), 4.48 (qd, J_1 = 7.1 Hz, J_2 = 4.5 Hz, 8H), 1.48 (td, J_1 = 7.1 Hz, J_2 = 3.4 Hz, 12H);^{13}C NMR (126 MHz, $CDCl_3$, ppm): δ (9 signals could not be detected due to signal overlap) 165.6, 165.3, 133.83, 133.79, 132.2, 132.1, 131.5, 131.3, 130.7, 61.8, 61.6, 32.1, 31.2;^{19}F NMR (376 MHz, $CDCl_3$, ppm): δ -122.5.

Synthesis of 5, 5'-(3-fluoropyridine-2, 5-diyl)diisophthalic acid (4)

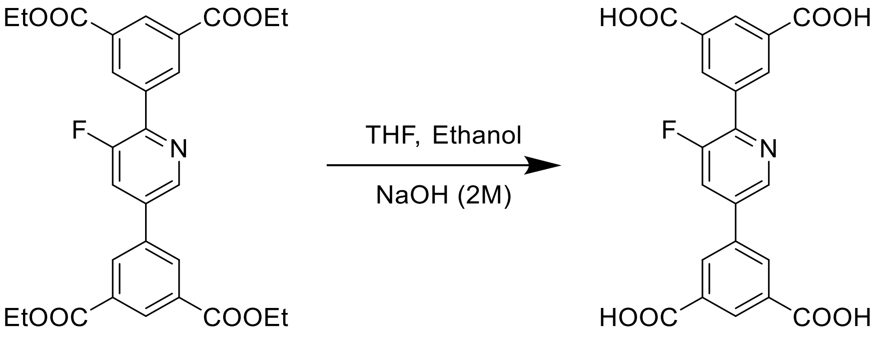

3 **4**

A mixture of **3** (0.5 g, 0.93 mmol), aqueous NaOH (2 M, 100 mL), EtOH (100 mL) and THF (100 mL) was refluxed for 17 h. The THF and EtOH were removed by evaporation and concentrated HCl added to the aqueous residue to pH ~1. The precipitate formed was collected by filtration, washed with H_2O and oven dried to afford the pure product **4** (0.39 g, 0.93 mmol, 99.6%) as a pale-yellow solid. ^{1}H NMR (400 MHz, DMSO-*d*$_6$, ppm): δ 8.41 (d, *J*=12.70 Hz, 1 H) 8.54 (s, 3 H) 8.57 (s, 1 H) 8.82 (s, 2 H) 9.03 (s, 1 H); ^{13}C NMR (126 MHz, DMSO-*d*$_6$, ppm): δ 166.8, 159.0, 156.9, 144.4, 142.3, 142.2, 136.6, 136.4, 135.7, 133.3, 133.0, 132.4, 132.2, 131.0, 130.5, 123.9, 123.7; ^{19}F NMR (376 MHz, DMSO-*d*$_6$, ppm): δ -123.2; MS (ESI): *m/z* calcd for $[C_{21}H_{12}NO_8F]^-$ – 424.31; found - 424.1.

Preparation of MFM-190(F)

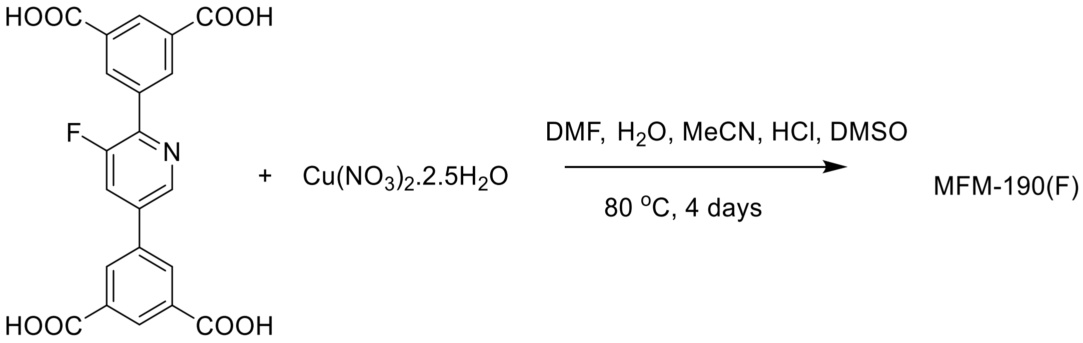

4

4 (0.1 g, 0.24 mmol) and $Cu(NO_3)_2.2.5H_2O$ (0.2 g, 1.07 mmol) were dissolved in a mixture of DMF (50 mL), H_2O (5 mL), MeCN (10 mL) and DMSO (5 mL) to which was added 1 mL of 1:1 *conc* HCl: DMF aqueous solution. The resulting mixture was then separated into ten pressure tubes, sealed and heated in an oil bath at 80°C for 4 days. The resulting deep blue crystals (yield ~42%) were then washed with DMF, acetone and dried in air. IR (cm^{-1}): 1141 and 926 (C-H), 900 (C-F). Elemental analysis (% calculated/found): C 43.1/43.2 H 2.1/2.0 F 3.3/3.4 N 2.4/2.3.

Synthesis of tetraethyl 5, 5'-(3-nitropyridine-2, 5-diyl)diisophthalate (6)

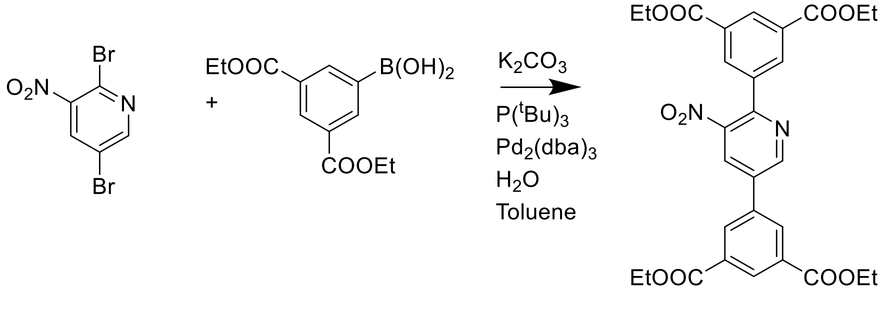

5 **2** **6**

A mixture of 2, 5-dibromo-3-nitropyridine (**5**) (1.41 g, 5.0 mmol), [3,5-bis(ethoxycarbonyl)phenyl]boronic acid (**2**) (3.19 g, 12.0 mmol), K_2CO_3 (3.36 g, 24.0 mmol), tri-*tert*-butylphosphine (3 mL), $Pd_2(dba)_3$ (0.89 g, 1.0 mmol), H_2O (85 mL) and toluene (350 mL) was heated at 80°C for 17 h and then at 90°C for 30 min under N_2 before cooling to room temperature. The reaction mixture was filtered, extracted with CH_2Cl_2 and the organic phase dried over $MgSO_4$. After filtration, the solvent was removed by evaporation and the residue purified by column chromatography using ethyl acetate/hexane (1:2) as eluent. The pure product **6** (2.15 g, 3.81 mmol, 76.2 %), was collected by filtration and dried in an oven, to afford a yellow solid. ^1H NMR (500 MHz, $CDCl_3$, ppm): δ 9.12 (d, J = 1.7 Hz, 1H), 8.74 (m, two almost resolved singlets (~1:1 ratio), 2H), 8.47 (m, two overlapped singlets, 3H), 8.38 (s, 2H), 4.4 (m, two overlapping quartets (~1:3:3:2:3:3:1 ratio), J = 7.17 Hz, 8H), 1.38 (dt, J_1 = 17.6 Hz, J_2 = 7.1 Hz, 12H); ^{13}C NMR (126 MHz, $CDCl_3$, ppm): δ (8 signals could not be detected due to signal overlap and noise) 165.2, 150.7, 133.5, 132.6, 132.2, 61.8, 14.5.

Synthesis of 5, 5'-(3-nitropyridine-2, 5-diyl)diisophthalic acid (7)

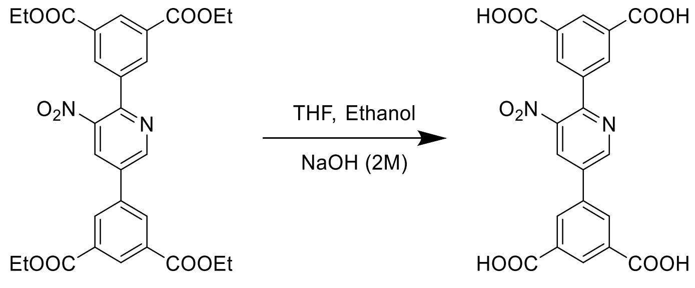

6 **7**

A mixture of **6** (2.2 g, 3.8 mmol), aqueous NaOH (2 M, 200 mL), ethanol (200 mL) and THF (200 mL) was refluxed for 17 h. The THF and EtOH were removed by evaporation and concentrated HCl added to the aqueous residue to pH ~1. The precipitate formed was collected by filtration, washed with H_2O and oven dried to afford the pure product **7** (1.72 g, 3.80 mmol, 99.7%) as a pale-yellow solid. ^1H NMR (400 MHz, DMSO-d_6, ppm): δ 13.6 (broad, s, 4H), 9.41 (s, 1H), 8.97 (s, 1H), 8.62 (s, 2H), 8.59 (m, 2H), 8.39 (s, 2H); ^{13}C NMR (126 MHz, DMSO-d_6, ppm): δ 166.8, 166.5, 151.3, 150.1, 149.1, 146.4, 136.0, 134.9, 133.2, 133.0, 132.6, 132.4, 131.9; MS (ESI): *m/z* calcd for $[C_{21}H_{12}N_2O_{10}]^-$ - 451.33; found – 451.3.

Preparation of MFM-190(NO_2)

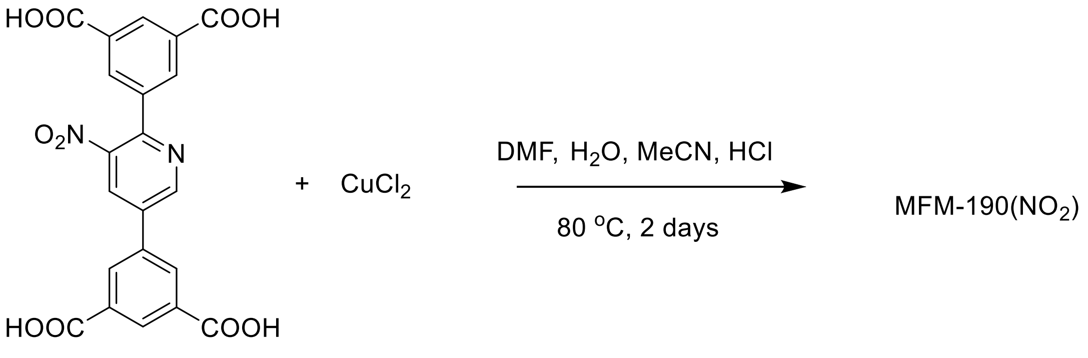

7

7 (0.10 g, 0.22 mmol) and $CuCl_2$ (0.18 mg, 1.34 mmol) were dissolved in a mixture of DMF (17 mL), H_2O (9 mL) and MeCN (6 mL) to which was added 1.2 mL of 2 M HCl (diluted from conc. HCl with DMF) aqueous solution. The resulting mixture was placed into a pressure tube, sealed and heated in an oil bath at 80°C for 2 days. The resulting green crystals (yield ~40%) were then washed with DMF, acetone and dried in air. IR (cm^{-1}): 1659 (C=O), 1382 and 728 (C-H), 1256 and 1089 (C-O). Elemental analysis (% calculated/found): C 41.3/41.1 H 2.0/2.0 N 4.6/4.3.

Synthesis of tetramethyl 5, 5'-(3-methylpyridine-2, 5-diyl)diisophthalate (10)

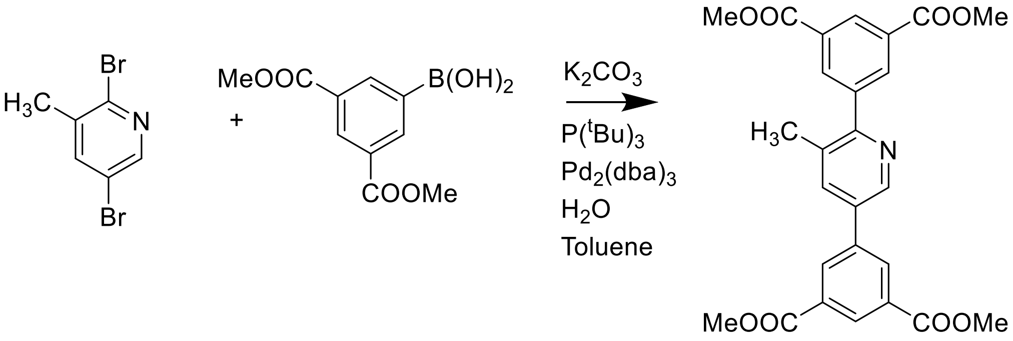

8 **9** **10**

A mixture of 2,5-dibromo-3-methylpyridine (**8**) (1.27 g, 5.0 mmol), [3,5-bis(methoxycarbonyl)phenyl]boronic acid (**9**) (2.86 g, 12.0 mmol), K_2CO_3 (3.36 g, 24.0 mmol), tri-*tert*-butylphosphine (3 mL), $Pd_2(dba)_3$ (0.89 g, 1.0 mmol), H_2O (85 mL) and toluene (350 mL) was heated at 80°C for 17 h and then at 90°C for 30 min under N_2 before cooling to room temperature. The reaction mixture was filtered, extracted with CH_2Cl_2 and the organic phase dried over $MgSO_4$. After filtration, the solvent was removed by evaporation and the residue purified by recrystallisation with dichloromethane and methanol. The pure product **10** (1.69 g, 3.54 mmol, 70.7 %), was collected by filtration and dried in an oven, to afford a white solid. ^1H NMR (500 MHz, $CDCl_3$, ppm): δ 8.78 (d, J = 1.5 Hz, 1H), 8.69 (t, J = 1.5 Hz, 1H), 8.66 (t, J = 1 Hz, 1H), 8.44 (d, J = 1.4 Hz, 2H), 8.41 (d, J = 1.5 Hz, 2H), 7.83 (d, J = 1.4 Hz, 1H), 3.93 (d, J = 15 Hz, 12H), 2.42 (s, 3H); ^{13}C NMR (126 MHz, $CDCl_3$, ppm): δ 166.1, 166.0, 156.2, 145.6, 140.8, 138.3, 137.3, 134.4, 133.8, 132.2, 131.6, 131.3, 130.8, 130.4, 130.2, 52.6, 52.5, 20.1.

Synthesis of 5, 5'-(3-methylpyridine-2, 5-diyl)diisophthalic acid (11)

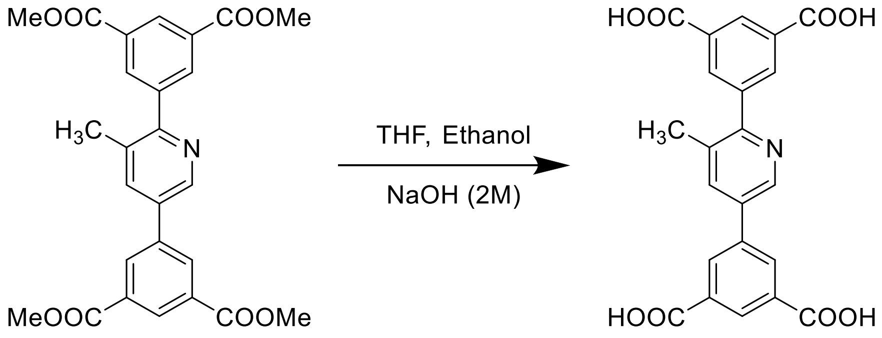

10 **11**

A mixture of **10** (1.5 g, 3.14 mmol), aqueous NaOH (2 M, 150 mL), EtOH (150 mL) and THF (150 mL) was refluxed for 17 h. The THF and EtOH were removed by evaporation and concentrated HCl added to the aqueous residue to pH ~1. The precipitate formed was collected by filtration, washed with H_2O and oven dried to afford the pure product **11** (1.32 g, 3.13 mmol, 99.7%) as an off-white solid. ^{1}H NMR (500 MHz, DMSO-d_6, ppm): δ 13.49 (broad s, 4H), 8.97 (s, 1H), 8.59 (s, 1H), 8.56 (s, 1H), 8.55 (s, 2H), 8.46 (s, 2H), 8.25 (s, 1H), 2.53 (s, 3H); ^{13}C NMR (126 MHz, DMSO-d_6, ppm): δ 166.90, 166.86, 155.6, 145.7, 140.9, 138.1, 137.9, 134.2, 133.5, 132.8, 132.0, 131.8, 131.7, 129.9, 20.0; MS (ESI): *m/z* calcd for $[C_{22}H_{15}NO_8]^-$ - 420.34; found – 420.1.

Preparation of MFM-190(CH_3)

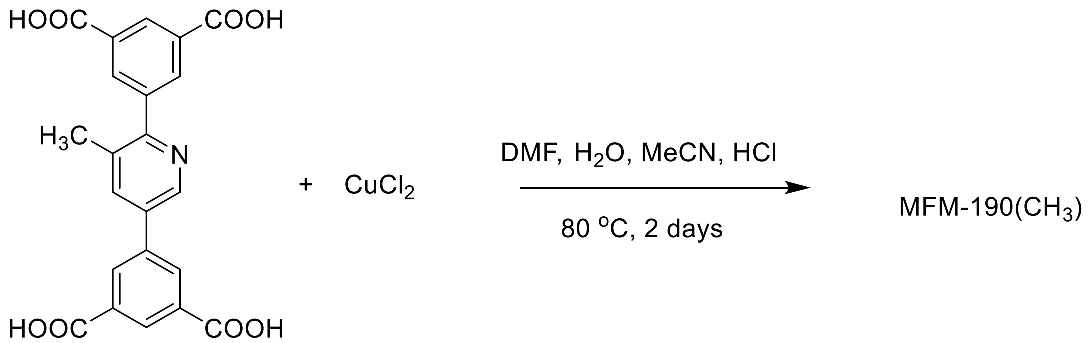

11

11 (0.20 mg, 0.48 mmol) and $CuCl_2$ (0.38 mg, 2.83 mmol) were dissolved in a mixture of DMF (34 mL), H_2O (18 mL) and MeCN (12 mL), to which was added 2.4 mL of 2 M HCl (diluted from *conc*. HCl with DMF) aqueous solution. The resulting mixture was placed into a pressure tube, sealed and heated in an oil bath at 80°C for 2 days. The resulting blue rhombus shaped crystals (yield ~45%) were then washed with DMF. IR (cm^{-1}): 1633 (C=O), 1363 and 727 (C-H), 1099 (C-O). Elemental analysis (% calculated/found): C 45.5/45.1 H 2.6/2.4 N 2.4/2.3.

Synthesis of MFM-100, MFM-101, MFM-102, MFM-190(H) (ZJU-5) and MFM-126

MFM-100, MFM-101, MFM-102, MFM-190(H) (ZJU-5) and MFM-126 were prepared according to literature procedures.[1-3]

Single crystal X-ray analysis

Integration, absorption correction, and determination of unit cell parameters were performed using the CrysAlisPro program package (CrysAlisPro 1.171.40.14a Rigaku Oxford Diffraction 2018). The structures were solved by dual space algorithm[4] and refined by the full-matrix least squares technique[5] in the anisotropic approximation (except hydrogen atoms). Positions of hydrogen atoms of organic ligands were calculated geometrically and refined in the riding model. The SQUEEZE[6] procedure was applied to account for the electron density within the pores. The crystallographic data and details of the structure refinements are summarized in Table S1.

Table S1. Summary of crystallographic data of MFM-190(F), MFM-190(NO_2) and MFM-190(CH_3)

	**MFM-190(F)**	**MFM-190(NO_2)**	**MFM-190(CH_3)**
Formula	$C_{21}H_{12}Cu_2FNO_{10}$	$Cu_2C_{21}H_{12}NO_{12}$	$Cu_2C_{22}H_{15}NO_{10}$
Formula Weight	584.42	611.41	580.43
Crystal System	Trigonal	Trigonal	Trigonal
Space group	$R\bar{3}m$	$R\bar{3}m$	$R32$
a, Å<br>c, Å	18.5907(2)<br>38.4499(5)	18.1941(4)<br>39.3568(14)	18.4874(1)<br>38.7587(4)
V, Å^3	11508.5(3)	11282.7(7)	11472.3(2)
Z	9	9	9
D_c, g cm^{-3}	0.759	0.810	0.756
μ, mm^{-1}	0.793	1.342	0.793
Crystal size, mm	0.01 x 0.03 x 0.06	0.03 x 0.03 x 0.05	0.01 x 0.03 x 0.05
Temperature, K	120	150	120
Radiation wavelength, Å	0.68890	CuKα 1.54184	0.68890
θ range, °	1.6 - 24.5	3.0 - 70.9	1.6 - 36.3
Reflections collected/ unique	10319, 2574	11482, 2652	84424, 12896,
Reflections with $I > 2\sigma$(I)	2350	2313	8873
R_{int}	0.062	0.027	0.097
$R[F^2 > 2\sigma(F^2)]$, $wR(F^2)$, S	0.0538, 0.1789, 1.09	0.0393, 0.1243, 1.09	0.0416, 0.1126, 0.90
Number of reflections	2574	2652	12896
Number of parameters	104	146	193
$\Delta\rho_{\text{max}}$/ $\Delta\rho_{\text{min}}$,eÅ^{-3}	-0.46, 0.69	-0.28, 0.62	-0.29, 0.94
CCDC Deposit Number	1863380	1863378	1863379

Crystallographic data for the reported structures have been deposited to the Cambridge Crystallographic Data Centre under the reference numbers CCDC 1863380, 1863378 and 1863379 for MFM-190(F), MFM-190(NO_2), and MFM-190(CH_3) respectively.

Powder X-ray diffraction

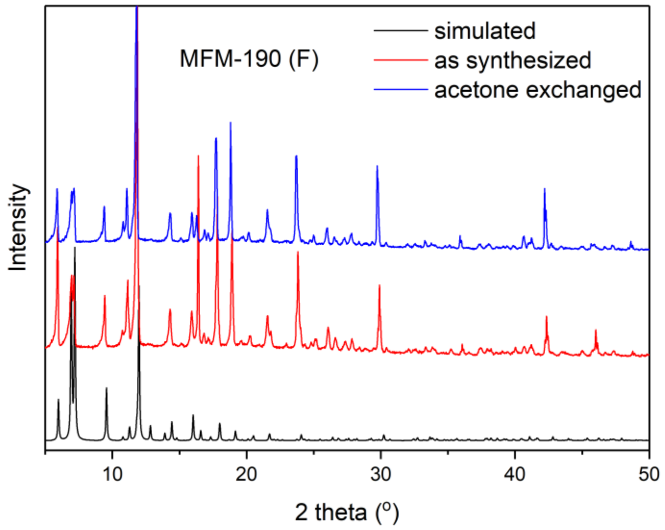

Fig. S1. PXRD patterns for the as-synthesized and acetone exchanged MFM-190(F) compared with simulation from the cif file. Calculated PXRD patterns were generated using Mercury 3.10.

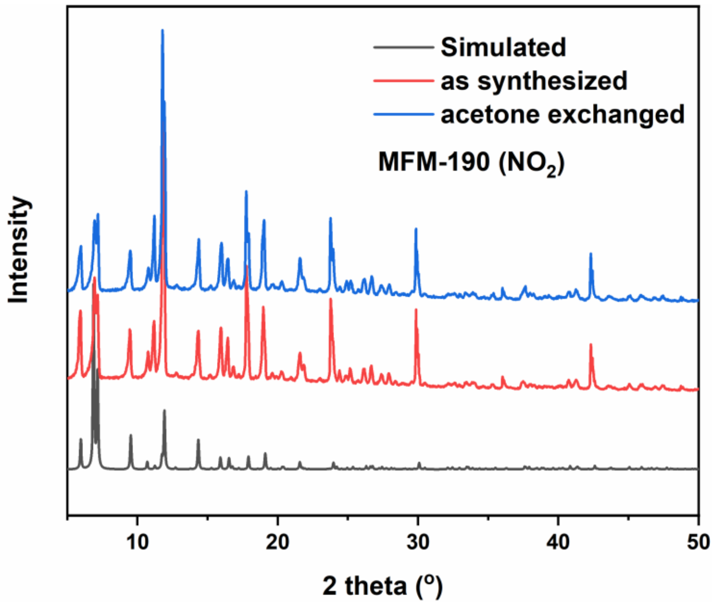

Fig. S2. PXRD patterns for the as-synthesized and acetone exchanged MFM-190(NO_2) compared with simulation from the cif file. Calculated PXRD patterns were generated using Mercury 3.10.

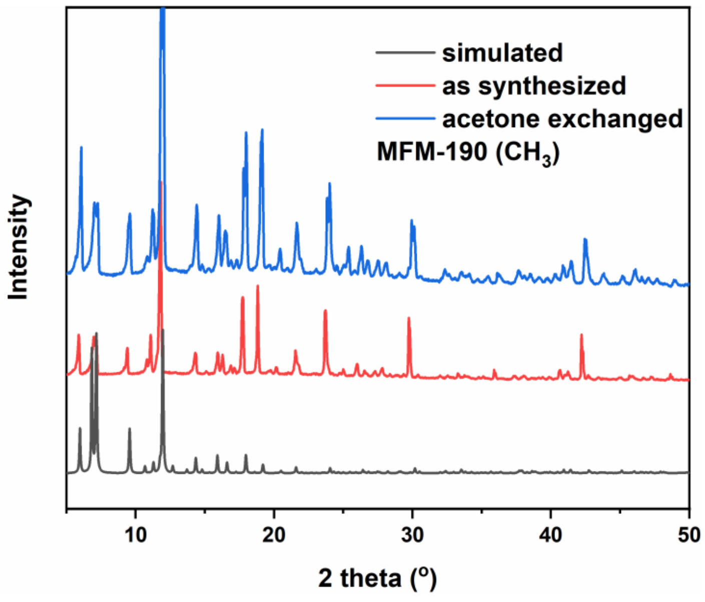

Fig. S3. PXRD patterns for the as-synthesized and acetone exchanged MFM-190(CH_3) compared with simulation from the cif file. Calculated PXRD patterns were generated using Mercury 3.10.

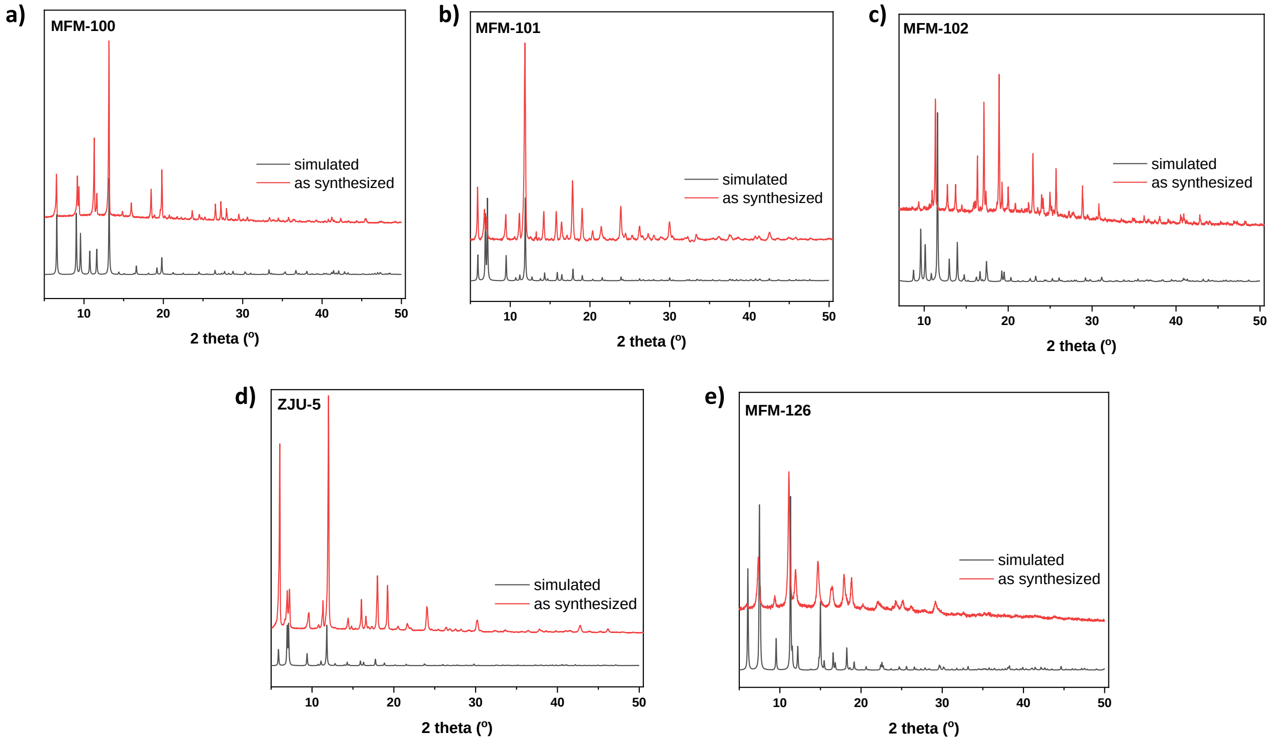

Fig. S4. PXRD patterns for the as-synthesized and simulations from cif files for a) MFM-100, b) MFM-101, c) MFM-102, d) ZJU-5 [MFM-190(H)] and e) MFM-126 and. Calculated PXRD patterns were generated using Mercury 3.10.

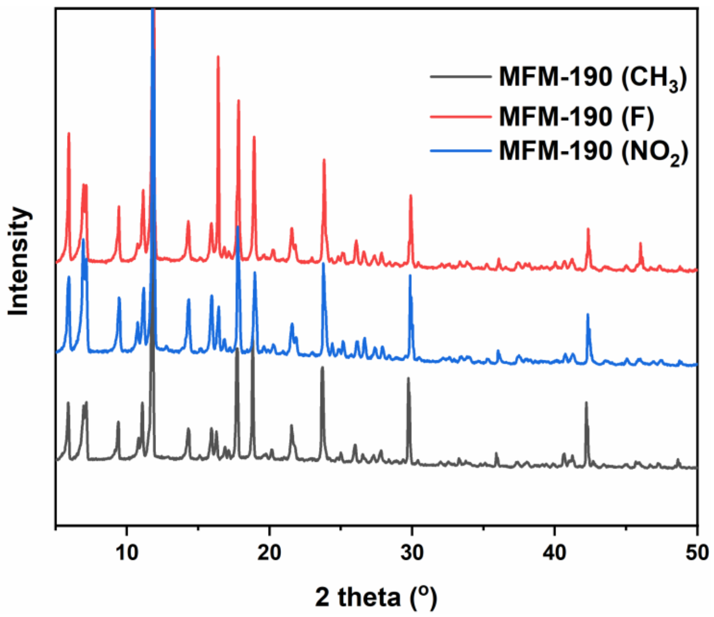

Fig. S5. Comparison of the PXRD patterns for MFM-190(F), MFM-190(NO_2) and MFM-190(CH_3).

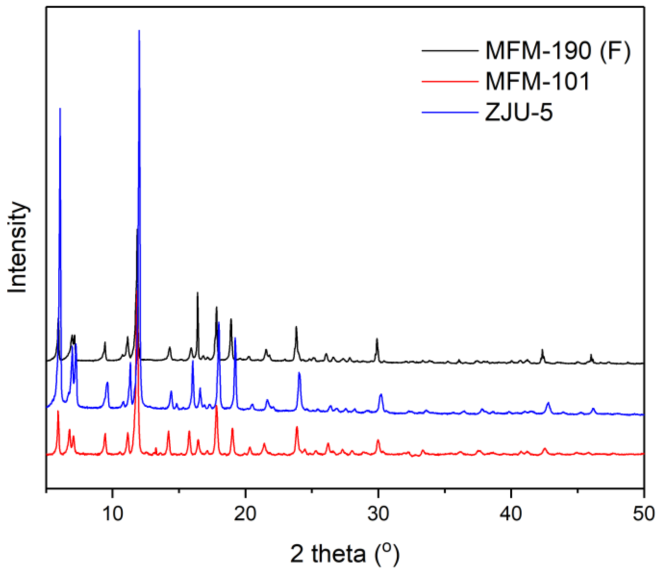

Fig. S6. Comparison of the PXRD patterns for MFM-190 (F), MFM-101 and ZJU-5 [MFM-190(H)].

Thermo-gravimetric analysis

As-synthesized samples of MFM-190(F), MFM-190(NO_2) and MFM-190(CH_3) were analysed by thermo-gravimetric analysis (TGA). MFM-190(F), MFM-190(NO_2) and MFM-190(CH_3) demonstrate similar thermal stability with the framework stable towards temperatures of up to ~300°C. The samples were first removed from their mother liquor and dried *via* filtration. The initial loss of weight of ~5-20% from 25-100°C is therefore due to loss of uncoordinated water molecules. The next stage of up to ~300°C accounts for ~20% weight loss and is due to loss of coordinated and uncoordinated DMF and MeCN molecules. After 300°C, the sharp decline in weight % is due to decomposition of the organic linker molecules, and framework collapse.

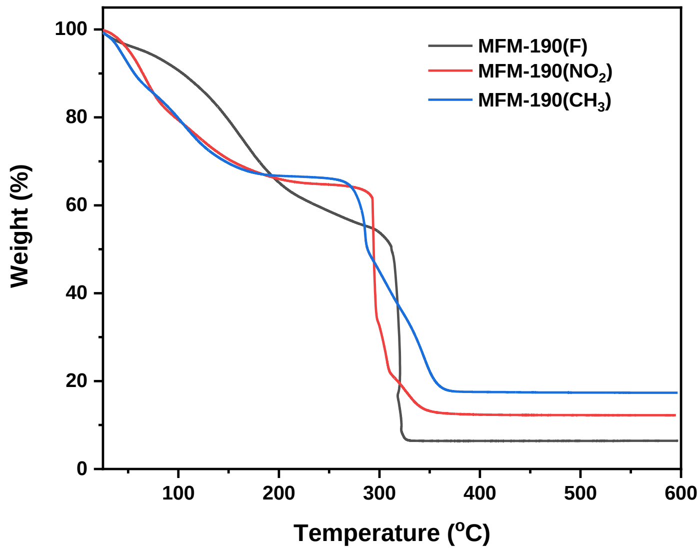

Fig. S7. TGA plots of as-synthesized samples of MFM-190(F), MFM-190(NO_2) and MFM-190(CH_3). The measurements were carried out under a flow of N_2 at a rate of 100 mL min^{-1}.

Characterisation of porosity and gas isotherms

Permanent porosity was established *via* low-pressure nitrogen sorption isotherms taken at 77 K of fully thermally activated, acetone exchanged samples. Ultra-high purity (99.999 %), CP grade N_2 was used as supplied by BOC.

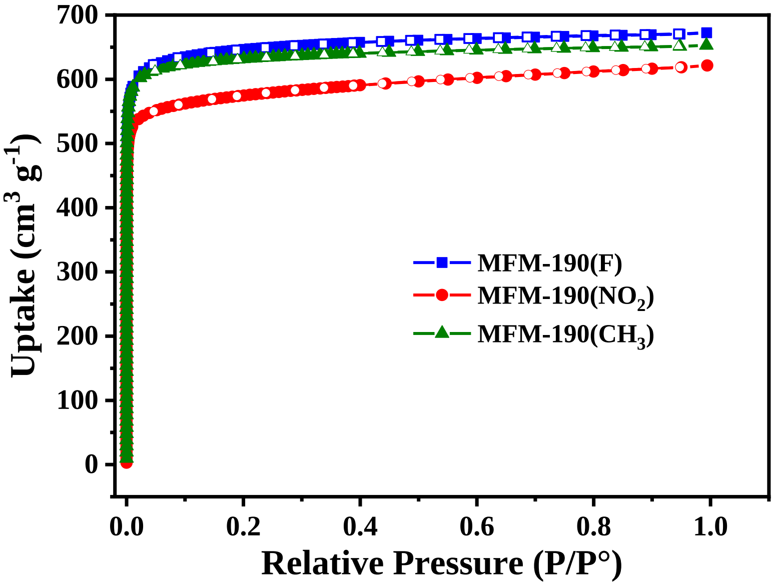

Fig. S8. N_2 adsorption isotherms at 77 K for MFM-190(F), MFM-190(NO_2) and MFM-190(CH_3).

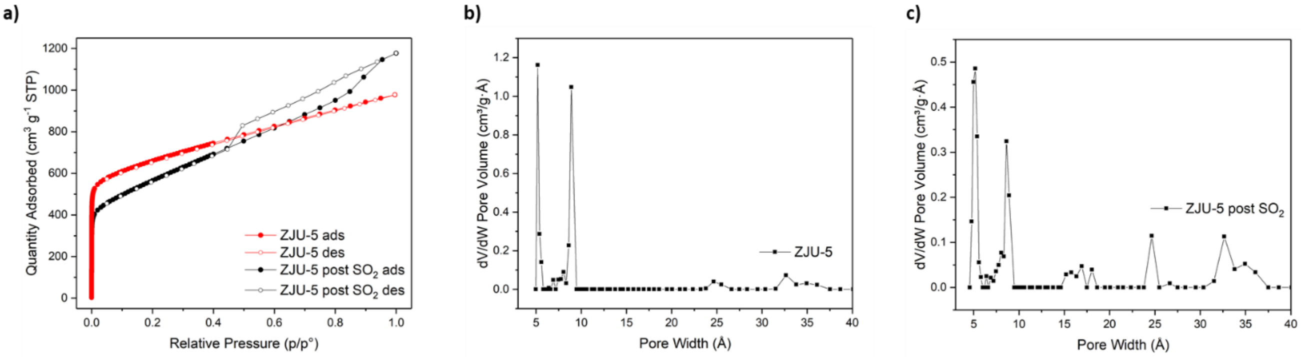

Fig. S9. a) N_2 isotherms at 77 K for ZJU-5 [MFM-190(H)] before and after sorption of SO_2 with visible hysteresis after exposure to SO_2. Pore size distributions (PSD) for ZJU-5 b) as-synthesized; c) after exposure to SO_2. The PSD were calculated from N_2 isotherms at 77 K using non-local density functional theory (NLDFT) based on a N_2-Carbon Finite, As = 6 model containing slit pores. As for the other MFM-190 series materials, as-synthesized ZJU-5 has a distribution of pores with estimated 5 Å and 7-9 Å.

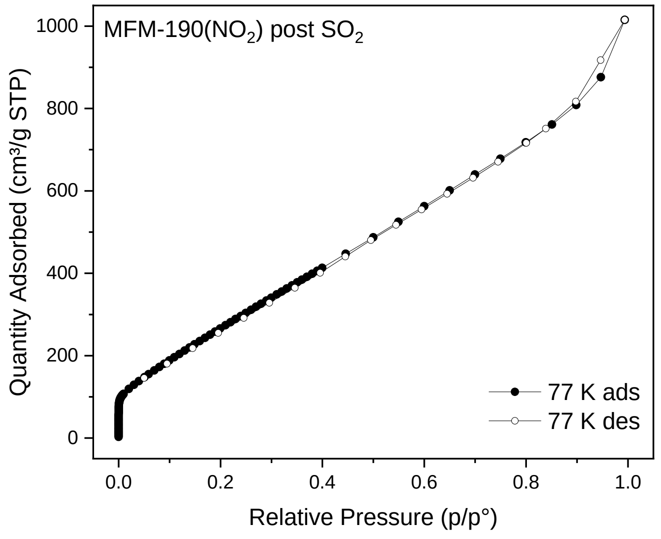

Fig. S10. N_2 isotherm at 77 K for MFM-190(NO_2) after exposure to SO_2.

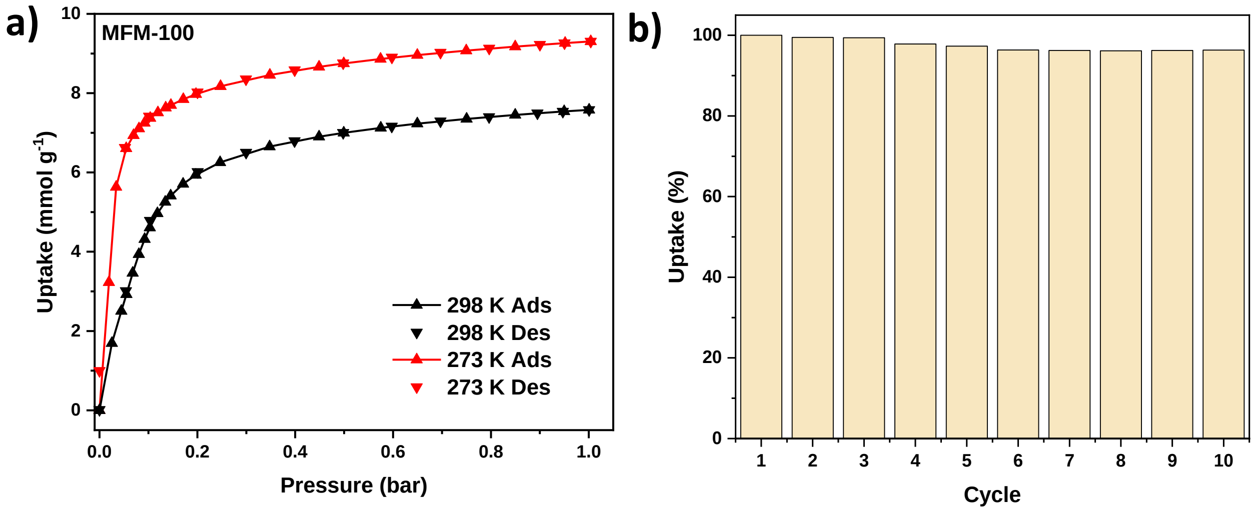

Fig. S11. a) Excess adsorption isotherms of SO_2 at 298 K and 273 K for MFM-100. b) Cycling of SO_2 between 0-500 mbar at 298 K in MFM-100.

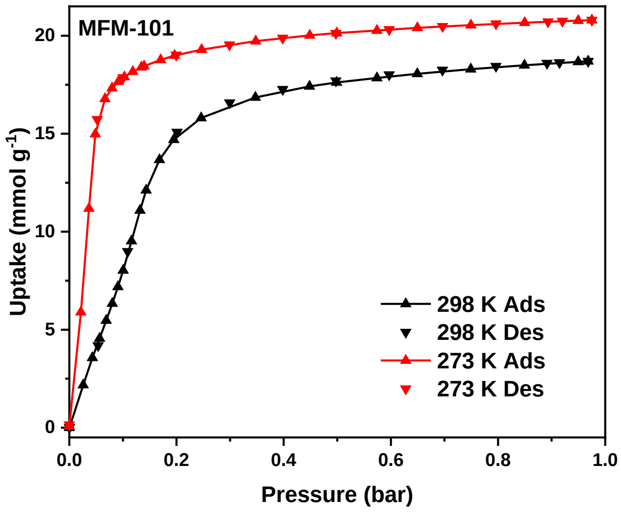

Fig. S12. Excess adsorption isotherms of SO_2 at 298 K and 273 K for MFM-101

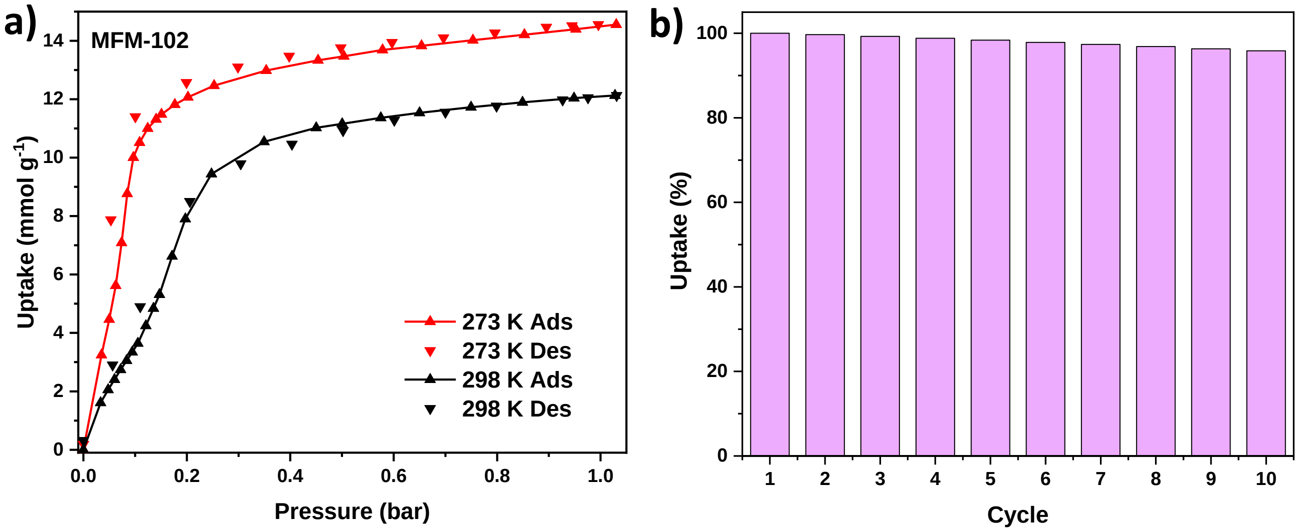

Fig. S13. a) Excess adsorption isotherms of SO_2 at 298 K and 273 K for MFM-102. b) Cycling of SO_2 between 0-500 mbar at 298 K in MFM-102.

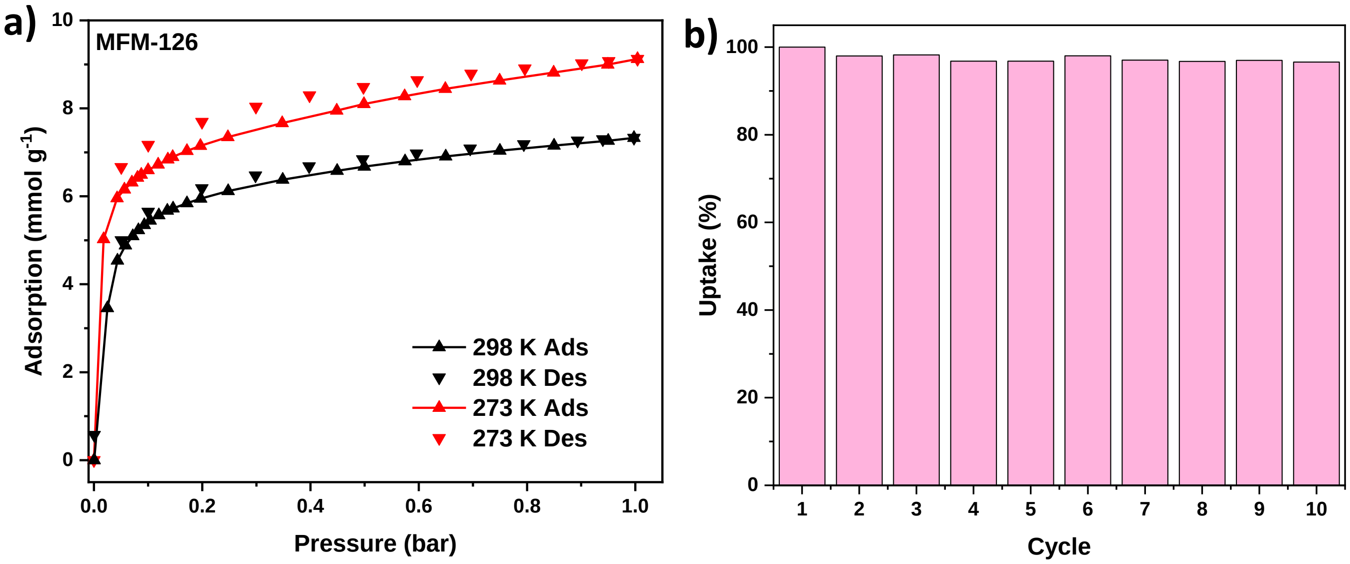

Fig. S14. a) Excess adsorption isotherms of SO_2 at 298 K and 273 K for MFM-126. b) Cycling of SO_2 between 0-500 mbar at 298 K in MFM-126.

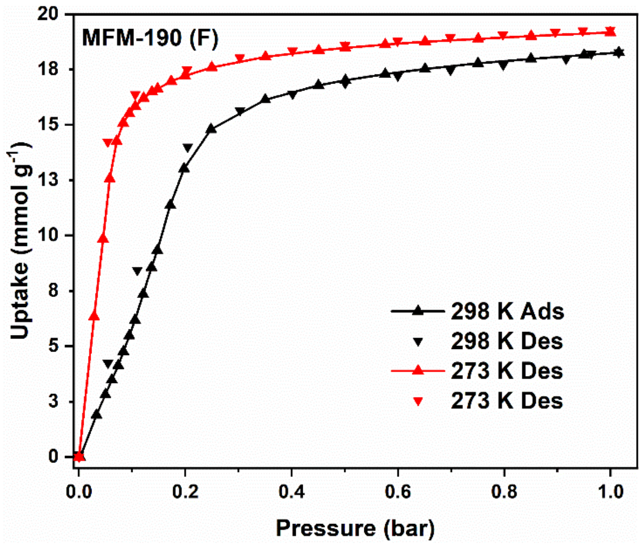

Fig. S15. Excess adsorption isotherms of SO_2 at 298 K and 273 K for MFM-190(F)

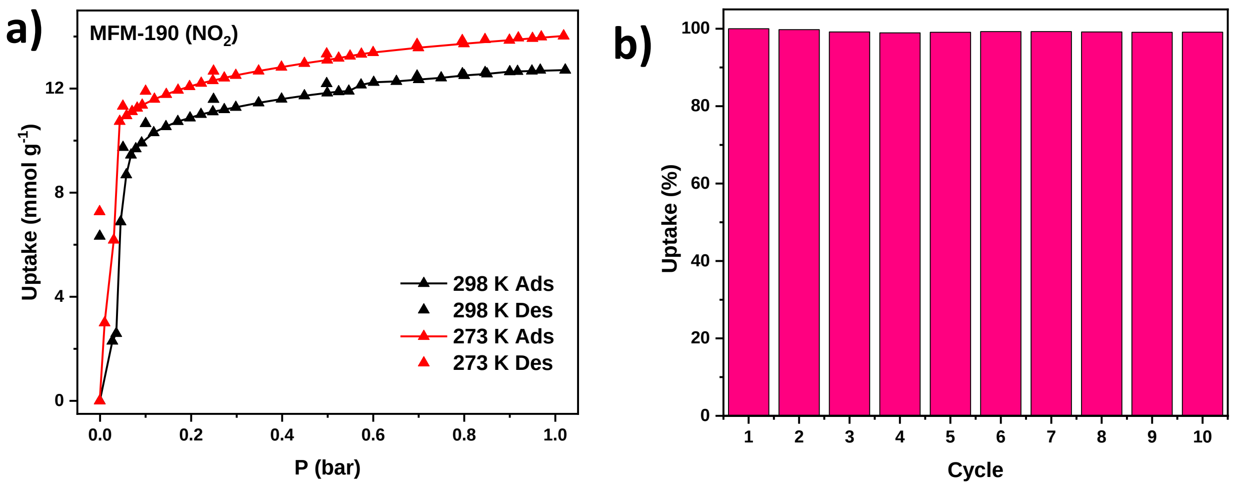

Fig. S16. a) Excess adsorption isotherms of SO_2 at 298 K and 273 K for MFM-190(NO_2). b) Cycling of SO_2 between 0-500 mbar at 298 K in MFM-190(NO_2).

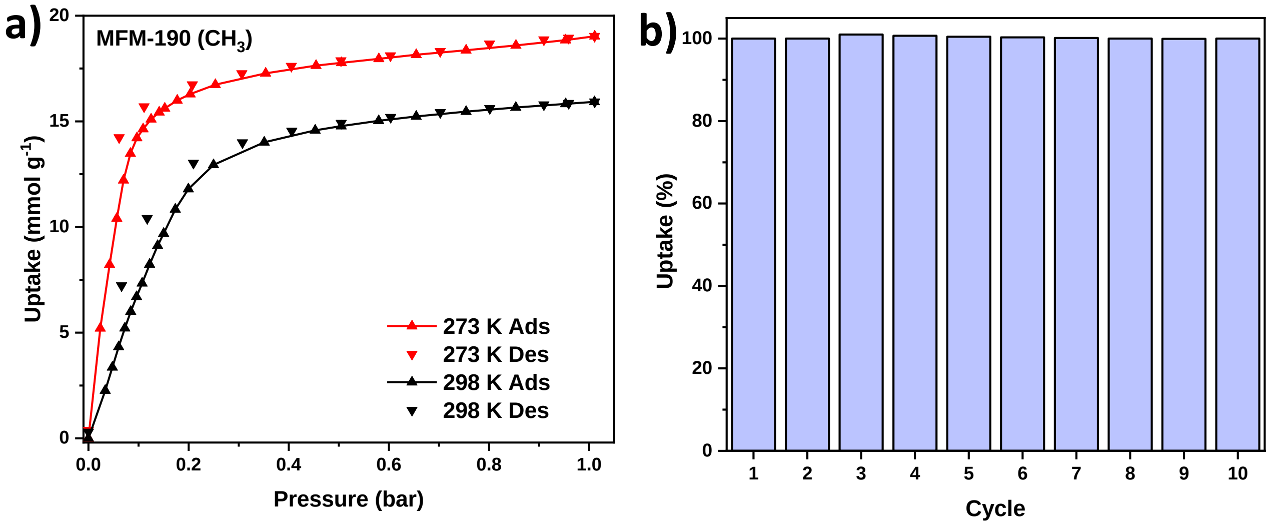

Fig. S17. a) Excess adsorption isotherms of SO_2 at 298 K and 273 K for MFM-190(CH_3). b) Cycling of SO_2 between 0-500 mbar at 298 K in MFM-190(CH_3).

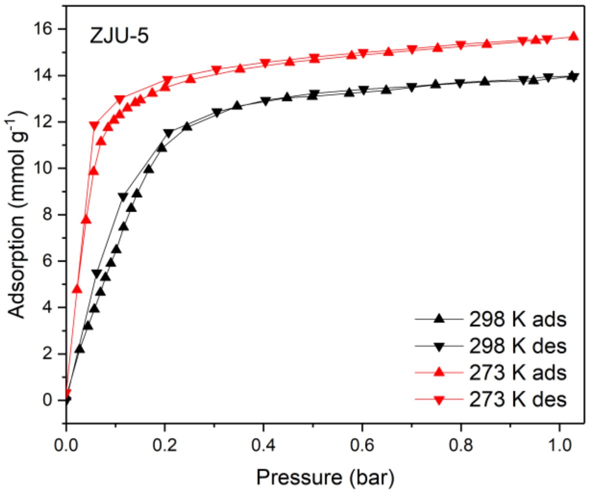

Fig. S18. Excess adsorption isotherms of SO_2 at 298 K and 273 K for ZJU-5 [MFM-190(H)].

PXRD of MOFs before and after SO_2 uptake

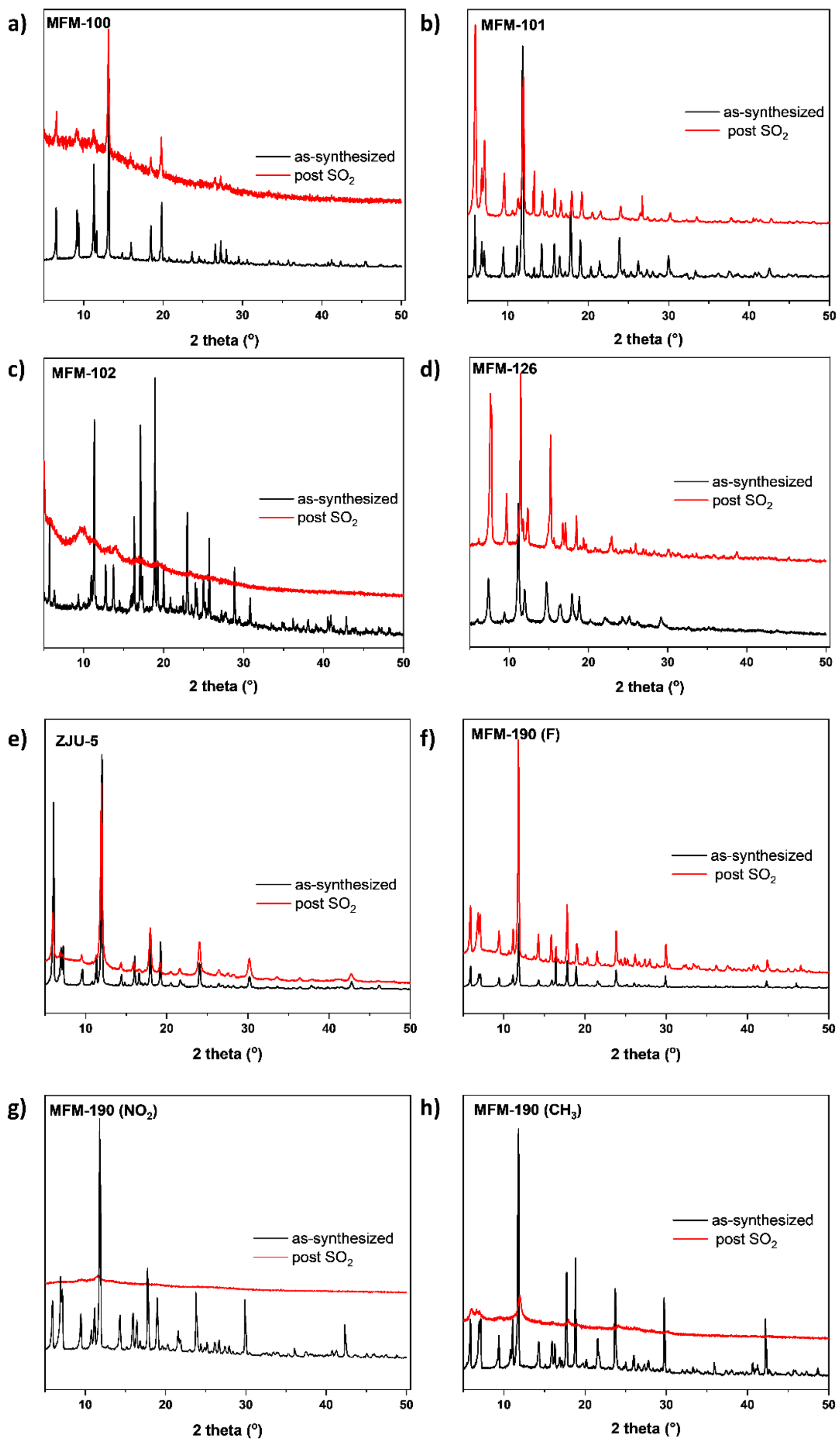

Fig. S19. PXRD patterns for the as-synthesized and post SO_2 exposure a) MFM-100, b) MFM-101 and c) MFM-102, d) MFM-126, e) ZJU-5 [MFM-190(H)], f) MFM-190(F), g) MFM-190(NO_2) and h) MFM-190(CH_3).

Dynamic breakthrough separation of SO_2 in MFM-190(F) and MFM-101

Breakthrough experiments were performed on a Hiden Isochema IGA-003 with ABR attachments and a Hiden Analytical mass spectrometer was used to detect the gases as they break through the sample bed. Experiments were carried out in a 7 mm diameter fixed-bed of 120 mm length packed with ~0.5 g of MFM-190(F) powder or ~0.3 g of MFM-101 powder (particle size < 1 micron). The sample was heated at 373 K under a flow of He for 12 h to activate the sample. The fixed-bed was cooled to room temperature (298 K) using a temperature programmed water bath and the breakthrough experiment performed with a stream of 0.5% SO_2 (diluted in He) at atmospheric pressure and room temperature. The flow rate of the entering gas mixture was maintained at 30 mL min^{-1}, and the gas concentration, C, of SO_2, CO_2 and N_2 at the outlet was determined by mass spectrometry and compared with the corresponding inlet concentration C_0, where $C/C_0 = 1$ indicates complete breakthrough. Breakthrough separation of SO_2/CO_2 was conducted using a mixture of 0.25% SO_2 (2500 ppm) and 15% CO_2 (*v/v*) diluted in He and N_2 through a fixed-bed packed with MFM-190(F) or MFM-101 at 298 K and 1 bar at a flow rate of 30 mL min^{-1}. Breakthrough separation of SO_2/N_2 was conducted using a mixture of 0.25% SO_2 (2500 ppm) and 75% N_2 diluted in He through a fixed-bed packed with MFM-190 or MFM-101 at 298 K and 1 bar at a flow rate of 30 mL min^{-1}.

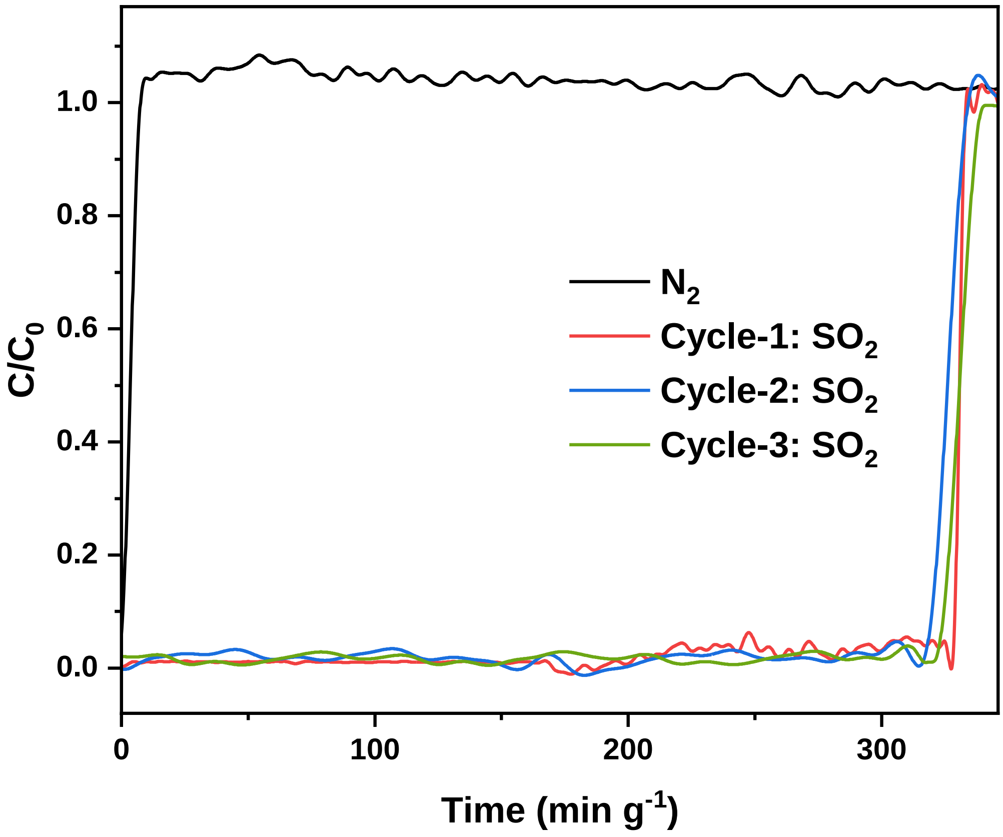

Fig. S20. Cyclic dynamic breakthrough plots for a gas mixture of SO_2/N_2 over a fixed-bed packed with MFM-190(F) (N_2/SO_2: 75%/2500 ppm, total flow rate 30 mL min^{-1}) at 298 K.

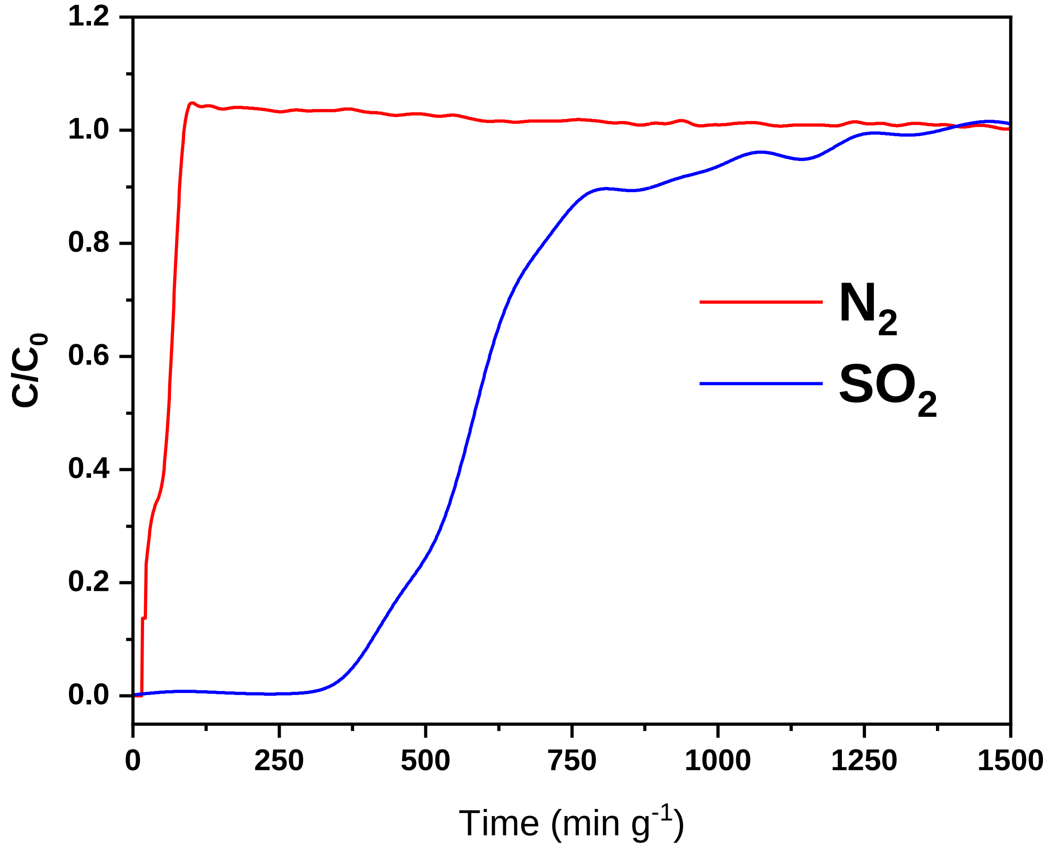

Fig. S21. Dynamic breakthrough plot for gas mixture of SO_2/N_2 in MFM-101 (N_2/SO_2: 75%/2500 ppm, total flow rate 30 mL min^{-1}) at 298 K.

Analysis of heat of adsorption

The interaction between SO_2 and the materials was analysed by coverage-dependent isosteric heats of adsorption (Q_{st}). The virial-type equation comprising the temperature-independent parameters a_i and b_j was employed to calculate the Q_{st} measured at 273 K and 298 K. In each case, the data were fitted using the equation below:

$$ln(P) = ln(N) + \left(\frac{1}{T}\right)\sum_{i=0}^{m} a_i N^i + \sum_{j=0}^{n} b_j N^j$$

The pressure, P, is expressed in mbar, and N is the amount of adsorbed in mol g^{-1}. T is the temperature in K, a_i and b_j are virial coefficients, and m, n represent the number of parameters required to sufficiently fit the isotherms (in this case, m and n were fixed at 4 and 3, respectively). The values of viral coefficients $a_0 - a_m$ were then used to estimate the Q_{st} using the following expression:

$$Q_{st} = -\mathrm{R}\sum_{i=0}^{m} a_i N^i$$

where R is the universal gas constant.

***In situ* FT-IR microscopy**

IR microscopic studies were carried out on MFM-101, MFM-190(F), MFM-190(H) and MFM-126 at the beamline B22 at the Diamond Light Source, Harwell UK. The instrument comprises a Bruker Hyperion 3000 microscope in transmission mode using a 15x IR objective and condenser with a liquid N_2 cooled 50 x 50 μm^2 MCT detector. This microscope is connected to a Bruker Vertex 80 V Fourier Transform IR interferometer using radiation generated from a bending magnet source. Spectra (256 scans) were collected in the range 500-4000 cm^{-1}, with 4 cm^{-1} resolution with an infrared spot size at the sample of approximately 25x25 μm. Acetone exchanged single crystals of MFM-101, MFM-190(F), MFM-190(H) and MFM-126 were loaded onto a ZnSe disk on a Linkam FTIR 600 sample cell, equipped with ZnSe windows and under temperature control. N_2 and SO_2 (as a function of partial pressure) were dosed into the cell using mass flow controllers at a constant total flow rate of 100 cm^3 min^{-1}. The samples were activated by heating to 383 K under flow of N_2, then cooled to 298 K and a background spectrum was collected. The samples were then dosed with increasing amounts of SO_2. Due to the low uptake capacity of MFM-126 for SO_2, the fundamental v_3 antisymmetric stretch of gas phase SO_2 at 1378 cm^{-1} saturates almost immediately (below 0.04 ppSO_2).

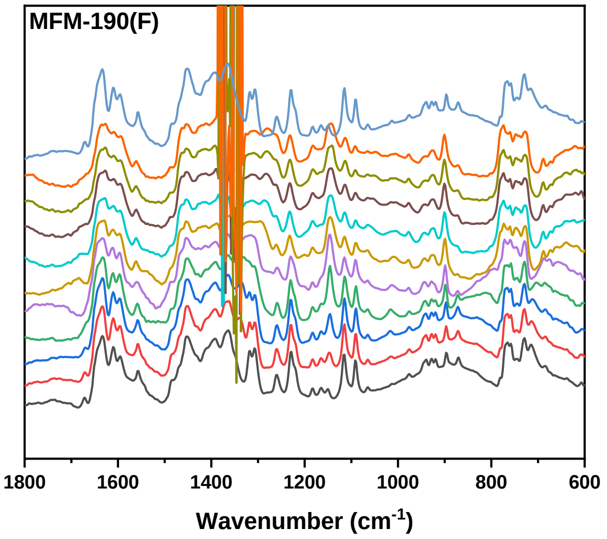

Fig. S22. *In situ* FT-IR spectra of MFM-190(F) as a function of SO_2 loading at 298 K. From the bottom to top: activated MFM-190(F), 1% SO_2, 2% SO_2, 5% SO_2, 10% SO_2, 20% SO_2, 40% SO_2, 60% SO_2, 80% SO_2, 100% SO_2 and regenerated MFM-190(F).

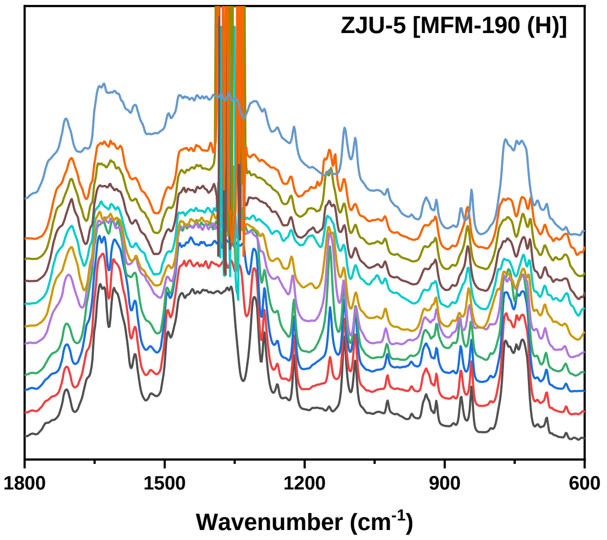

Fig. S23. *In situ* FT-IR spectra of ZJU-5 [MFM-190(H)] as a function of SO_2 loading at 298 K. From the bottom to top: activated ZJU-5, 1% SO_2, 2% SO_2, 5% SO_2, 10% SO_2, 20% SO_2, 40% SO_2, 60% SO_2, 80% SO_2, 100% SO_2 and regenerated ZJU-5.

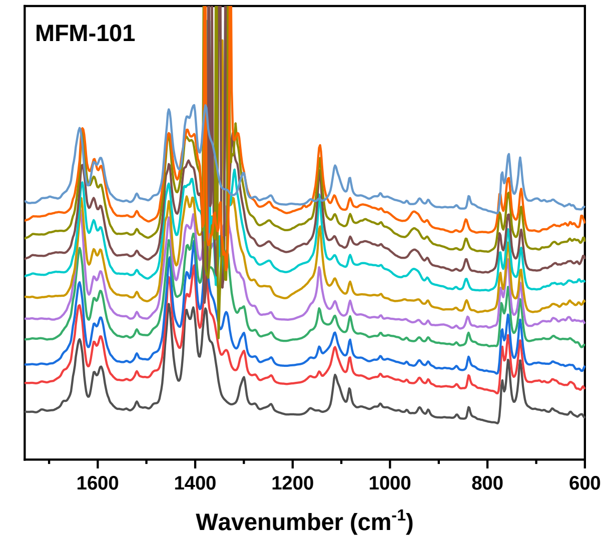

Fig. S24. *In situ* FT-IR spectra of MFM-101 as a function of SO_2 loading at 298 K. From the bottom to top: activated MFM-101, 1% SO_2, 2% SO_2, 5% SO_2, 10% SO_2, 20% SO_2, 40% SO_2, 60% SO_2, 80% SO_2, 100% SO_2 and regenerated MFM-101.

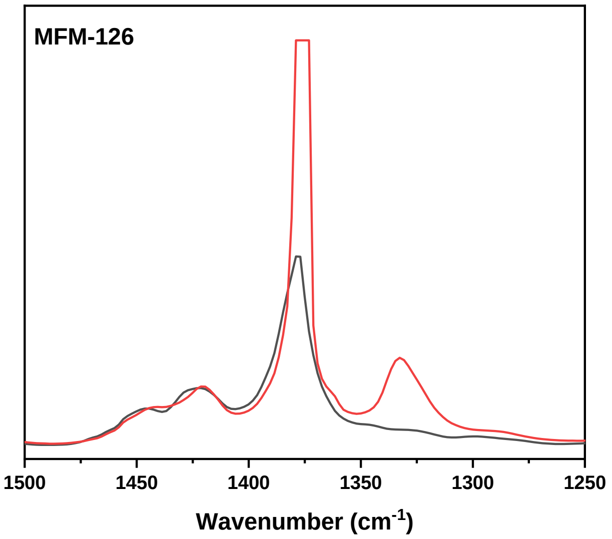

Fig. S25. *In situ* FT-IR of MFM-126 as a function of SO_2 loading at 298 K (activated MFM-126, black; 4% SO_2-loaded MFM-126, red). Saturation of the peak at 1378 cm^{-1}, assigned to the gas phase SO_2, appears at 4% SO_2 loading.

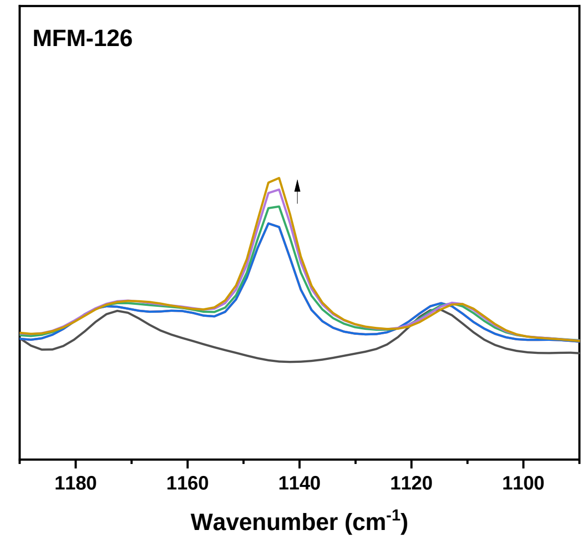

Fig. S26. *In situ* FT-IR of MFM-126 as a function of SO_2 loading at 298 K. From the bottom to top: activated MFM-126, 10% SO_2, 20% SO_2, 40% SO_2, 60% SO_2; the growth of a new peak at 1143 cm^{-1} is assigned to the ν_1 symmetric stretch of adsorbed SO_2.

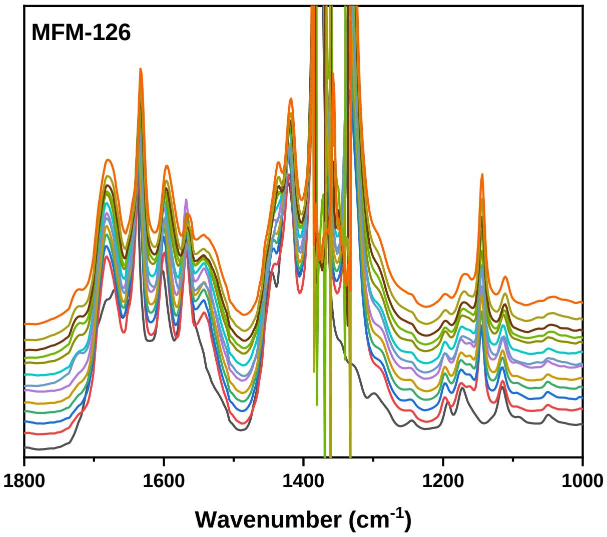

Fig. S27. *In situ* FT-IR spectra of MFM-126 as a function of SO_2 loading at 298 K. From the bottom to top: activated MFM-126, 2% SO_2, 4% SO_2, 6% SO_2, 8% SO_2, 10%, 12% SO_2, 16% SO_2, 20% SO_2, 40% SO_2, 60% SO_2, 80% SO_2, 100% SO_2.

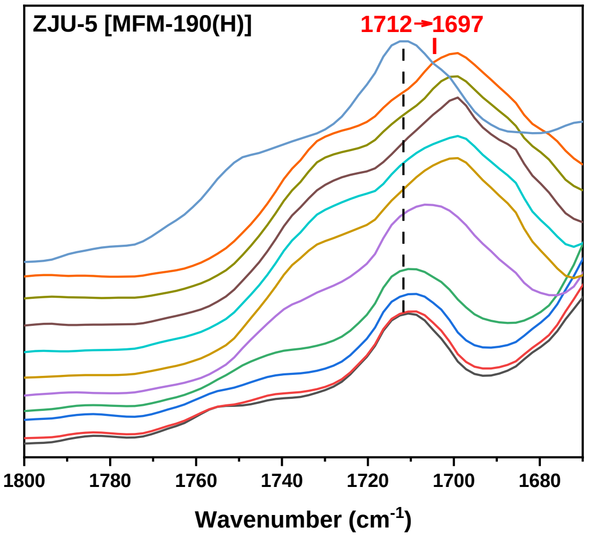

Fig. S28. *In situ* FT-IR spectra of ZJU-5 [MFM-190(H)] as a function of SO_2 loading at 298 K. From the bottom to top: activated ZJU-5, 1% SO_2, 2% SO_2, 5% SO_2, 10% SO_2, 20% SO_2, 40% SO_2, 60% SO_2, 80% SO_2, 100% SO_2 and regenerated ZJU-5.

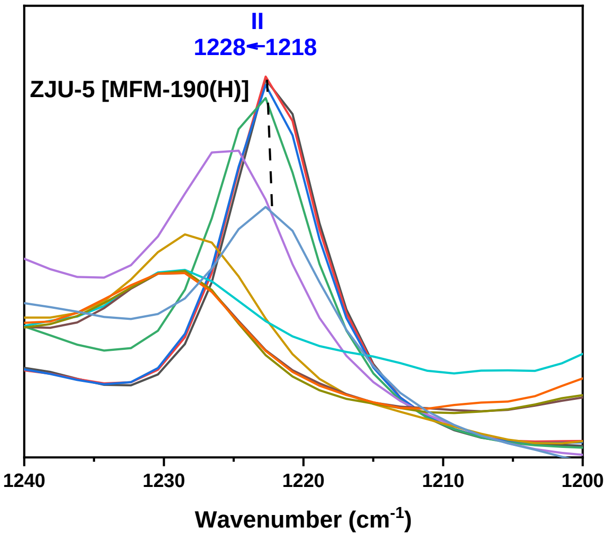

Fig. S29. *In situ* FT-IR spectra of ZJU-5 [MFM-190(H)] as a function of SO_2 loading at 298 K. From the bottom to top: activated ZJU-5, 1% SO_2, 2% SO_2, 5% SO_2, 10% SO_2, 20% SO_2, 40% SO_2, 60% SO_2, 80% SO_2, 100% SO_2 and regenerated ZJU-5.

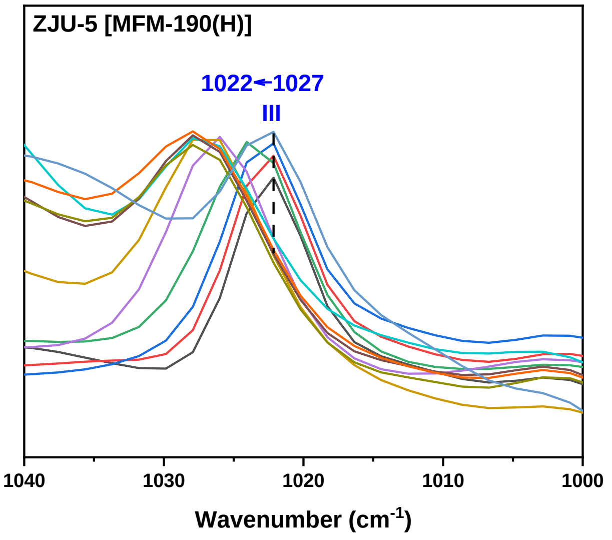

Fig. S30. *In situ* FT-IR spectra of ZJU-5 [MFM-190(H)] as a function of SO_2 loading at 298 K. From the bottom to top: activated ZJU-5, 1% SO_2, 2% SO_2, 5% SO_2, 10% SO_2, 20% SO_2, 40% SO_2, 60% SO_2, 80% SO_2, 100% SO_2 and regenerated ZJU-5.

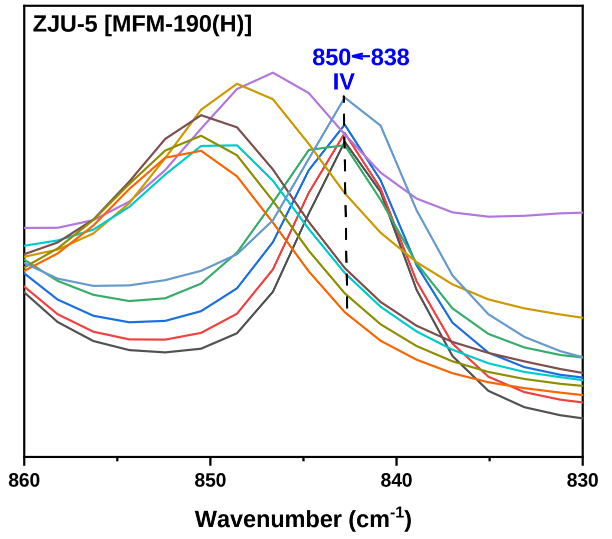

Fig. S31. *In situ* FT-IR spectra of ZJU-5 [MFM-190(H)] as a function of SO_2 loading at 298 K. From the bottom to top: activated ZJU-5, 1% SO_2, 2% SO_2, 5% SO_2, 10% SO_2, 20% SO_2, 40% SO_2, 60% SO_2, 80% SO_2, 100% SO_2 and regenerated ZJU-5.

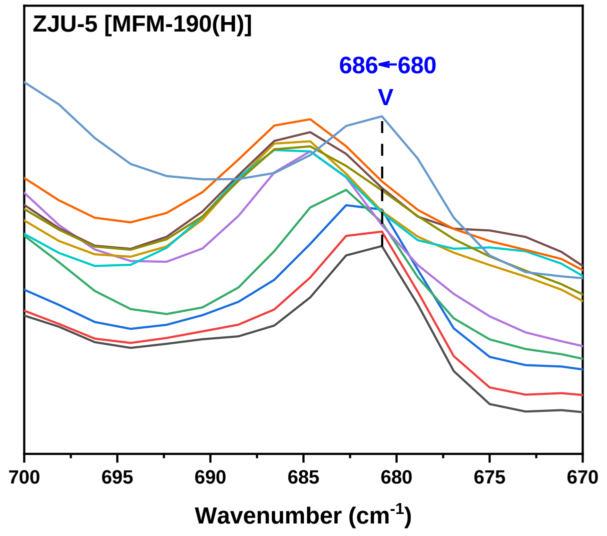

Fig. S32. *In situ* FT-IR spectra of ZJU-5 [MFM-190(H)] as a function of SO_2 loading at 298 K. From the bottom to top: activated ZJU-5, 1% SO_2, 2% SO_2, 5% SO_2, 10% SO_2, 20% SO_2, 40% SO_2, 60% SO_2, 80% SO_2, 100% SO_2 and regenerated ZJU-5.

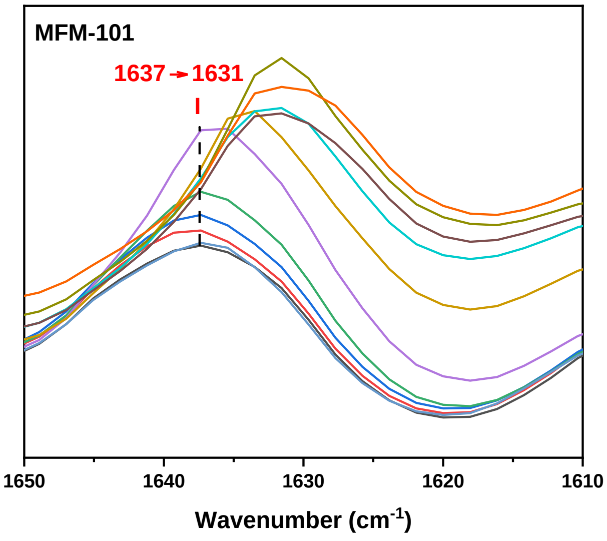

Fig. S33. *In situ* FTIR spectra of MFM-101 as a function of SO_2 loading at 298 K. From the bottom to top: activated MFM-101, 1% SO_2, 2% SO_2, 5% SO_2, 10% SO_2, 20% SO_2, 40% SO_2, 60% SO_2, 80% SO_2, 100% SO_2 and regenerated MFM-101.

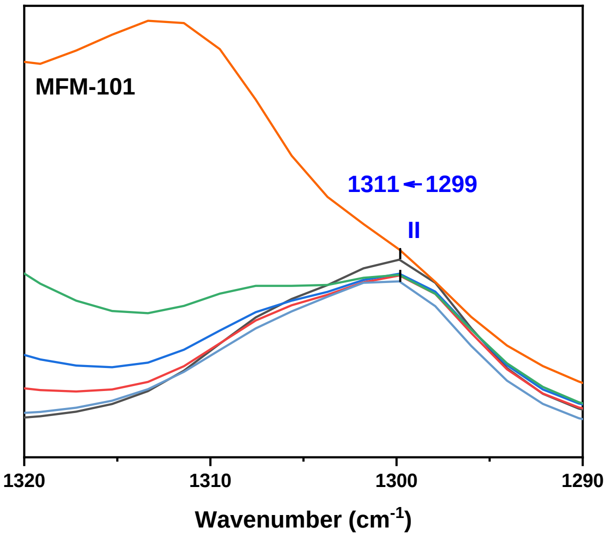

Fig. S34. *In situ* FT-IR spectra of MFM-101 as a function of SO_2 loading at 298 K. From the bottom to top: activated MFM-101, 1% SO_2, 2% SO_2, 5% SO_2, 100% SO_2 and regenerated MFM-101.

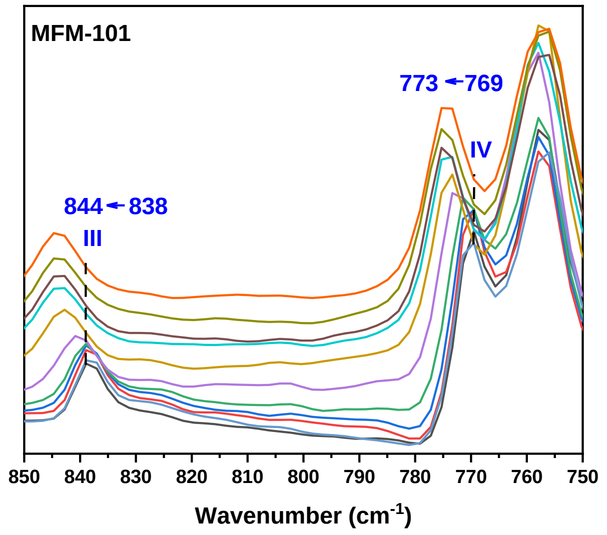

Fig. S35. *In situ* FT-IR spectra of MFM-101 as a function of SO_2 loading at 298 K. From the bottom to top: activated MFM-101, 1% SO_2, 2% SO_2, 5% SO_2, 10% SO_2, 20% SO_2, 40% SO_2, 60% SO_2, 80% SO_2, 100% SO_2 and regenerated MFM-101.

Rietveld refinement of NPD data

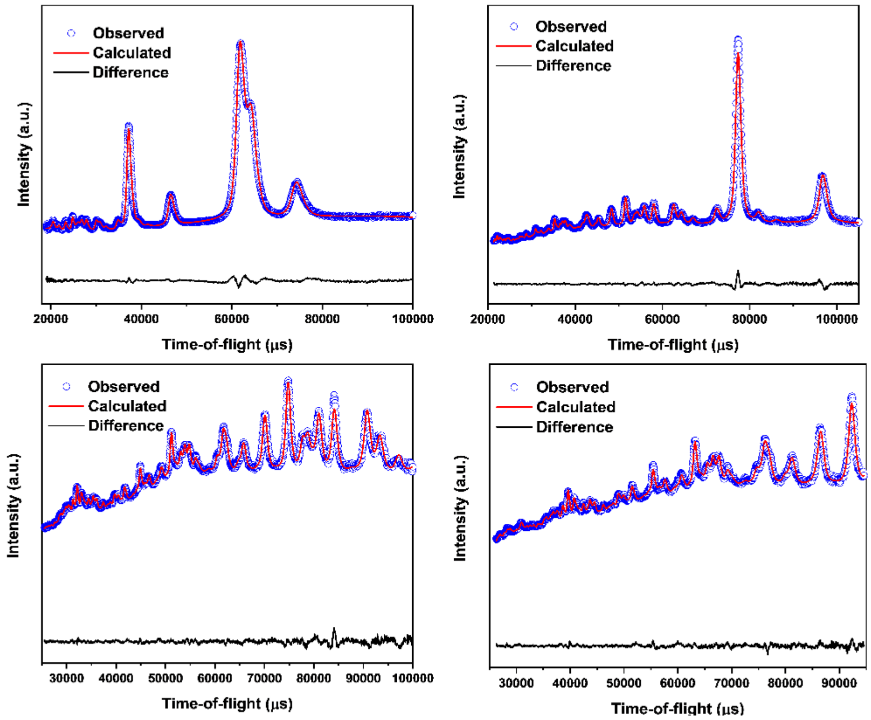

Fig. S36. NPD data [observed (blue), calculated (red) and difference (black)] for the Rietveld refinement of the $Cu_2(C_{21}H_{12}FNO_{10})\cdot(SO_2)_{3.2}$.

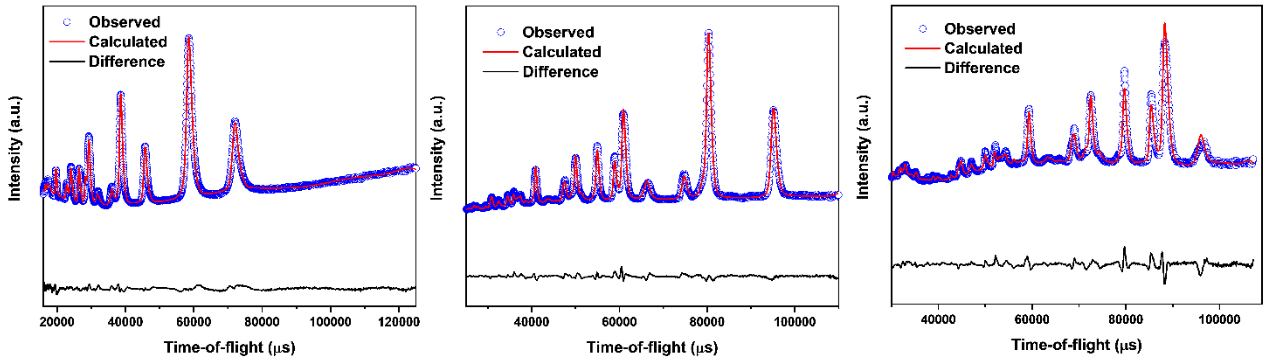

Fig. S37. NPD data [observed (blue), calculated (red) and difference (black)] for the Rietveld refinement of the $Cu_2(C_{35}H_{35}N_9O_{13})\cdot(SO_2)_{1.6}$.

Table S2. Details of crystal data for SO_2-loaded MFM-126 and MFM-190(F) determined by *in situ* neutron powder diffraction.

Sample	SO_2@MFM-126	SO_2@MFM-190(F)
Formula	$Cu_2(C_{35}H_{35}N_9O_{13})\cdot(SO_2)_{1.6}$	$Cu_2(C_{21}H_{12}FNO_{10})\cdot(SO_2)_{3.2}$
Crystal System	R-3m	R-3m
a, b (Å)	18.567(8)	18.556(5)
c (Å)	35.250(15)	38.530(4)
V [Å^3]	10524(10)	11490(2)
Radiation type	Neutron powder diffraction	Neutron powder diffraction
Scan method	Time of Flight	Time of Flight
Diffractometer	Beamline WISH at ISIS	Beamline WISH at ISIS
Temperature (K)	10	10
R_{exp} / %	0.290(8)	0.518(5)
R_{wp} / %	1.579(4)	0.936(9)
R_p / %	1.259(5)	0.877(4)
GoF	5.42(9)	1.80(6)
CCDC deposit number	2142215	2142216

The stability of MFM-190(F) toward water has been studied by PXRD. Unfortunately, MFM-190(F) suffers from structural degradation upon soaking in water, which is commonly observed for *nbo* MOFs.

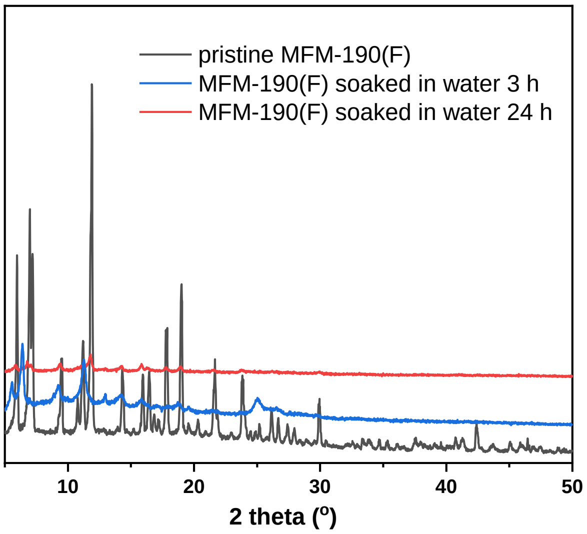

Fig. S38. PXRD patterns of MFM-190(F) upon soaking in water at room temperature.

Comparison of solid porous materials as sorbents for SO_2

Table S3 – Summary of SO_2 adsorption in various reported solid porous materials [a]

MOF	BET Surface Area (m^2 g^{-1})	Pore volume (cm^3 g^{-1})	SO_2 Adsorption Capacity (mmol g^{-1})	Selectivity for equimolar SO_2/CO_2	Open Metal Site	Reference
MFM-190(F)	2538	1.041	18.3	5.2[h]	Y	This work
MFM-190(NO_2)	2304	0.962	6.6*	-	Y	This work
MFM-190(CH_3)	2550	1.011	15.9*	-	Y	This work
ZJU-5	2373	1.002	14.0*	-	Y	This work
MFM-100	1445	0.68	7.6*	-	Y	This work
MFM-101	2300	0.885	18.7	2.5[h]	Y	This work
MFM-102	2873	1.138	12.1*	-	Y	This work
MFM-126	965	0.47	7.3	-	N	This work
MFM-170	2408	0.88	17.5	35	Y	7
MFM-170·H_2O	2003	n/a	13.0	-	N	7
SIFSIX-1-Cu	1337	n/a	11.0	71[f]	N	8
[$Zn_2(L_1)_2$(bipy)]	275	0.059	10.9	-	N	9
MFM-202a	2220	n/a	10.2	-	N	10
Ni(bdc)$(ted)_{0.5}$	1783	0.74	10.0	-	N	11
Mg-MOF-74	1475	0.62	8.6	-	Y	12
MFM-300(In)	1071	0.37	8.3	60	N	13
SIFSIX-2-Cu-i	735	n/a	6.9	87[f]	N	8
PI-COF-m	1003	n/a	6.5	-	n/a	14
SIFSIX-2-Cu	3140	n/a	6.5	-	N	8
[$Zn_2(L_1)_2$(bpe)]	379	0.081	6.4	-	N	9
PI-COF-m10	831	n/a	6.3	-	n/a	14
PI-COF-m20	548	n/a	5.6	-	n/a	14
PI-COF-m40	279	n/a	5.5	-	n/a	14
PI-COF-m60	93	n/a	4.7	-	n/a	14
Zn(bdc)$(ted)_{0.5}$	1888	0.84	4.4	-	N	11
SIFSIX-3-Ni	223	n/a	2.7	702[f]	N	8
Prussian Blue	712	n/a	2.5	-	N	15
[Zn4(μ_4-O)$(L_1)_3$]	299	0.047	2.2	-	N	9
FMOF-2	378	n/a	2.2	-	Y	16
SIFSIX-3-Zn	250	n/a	2.1	-	N	8
MFM-600	2281	n/a	5.0	13	N	17
MFM-601	3644	n/a	12.3	32	N	17
MFM-305	799	0.347	7.0	160	N	18
MFM-305-CH_3	256	0.209	5.2	160	N	18
KAUST-7	280	0.095	2.6	-	N	19
KAUST-8	250	0.102	2.9	-	Y	19
MIL-101(Cr)-4F(1%)	2176	1.19	18.4	-	Y	20
MFM-300(Sc)	1360	0.56	9.4	30	N	21
ECUT-100	-	0.27	5.0	27[g]	N	22
MFM-300(Sc)@EtOH	-	-	13.2	-	N	21

DMOF	2050	0.8	9.9	-	N	23
DMOF-TM	894	0.39	4.9	-	N	23
UR3-MIL-101(Cr)	1900	0.96	13.9[b]	-	n/a	24
MIL-101(Cr)	3300	1.49	18.4[b]	-	n/a	24
MFM-300(Cr)	1360	n/a	8.6[c]	79[c]	N	25
ECUT-111	1493	0.629	11.6	22[g]	N	26
Cage-U-Co-MOF	208	n/a	3.6	80[g]	Y	27
NH_2-MIL-101(Cr)	2290	1.16	16.7[b]	30	n/a	28
HKUST-1	1490	0.61	13.8[b]	28	Y	28
ZIF-8	1820	0.80	8.2[b]	-	N	28
ZIF-67	1980	0.69	11.0[b]	-	N	28
NOTT-401	1502	0.66	6.6	-	N	29
ECUT-77	760	0.40	8.0[d]	44[g]	N	30
NU-1000	1972	n/a	10.9	-	n/a	31
[Ir]@NU-1000	1858	n/a	10.6	-	n/a	31
CAU-10	630	0.25	4.5	-	N	32
CPL-1	335	0.125	2.0	-	N	33
UNAM-1	522	n/a	3.5[e]	-	N	34
ELM-12	706	0.26	2.7	30[f]	n/a	35
MOF-808	2380	1.1	15.3	8.9[f]	N	36
EDTA-MOF-808	1036	0.47	9.8	57.2[f]	N	36
[RuGa]@NU-1000	1796	n/a	7.5	-	N	37
CB_6@MIL-101-Cl	2077	1.0	17.0	-	n/a	38
MIL-53(Al)-TDC	1260	0.450	8.9[b]	-	N	39
MIL-53(Al)-BDC	1210	0.510	9.9[b]	-	N	39
Zr-Fum	600	0.290	4.9[b]	41[b]	n/a	40
DUT-67(Zr)	1260	0.544	9.0[b]	37[b]	n/a	40
MIL-53(Al)	1450	0.706	10.5[b]	43[b]	n/a	40
Al-Fum	970	0.447	7.5[b]	36[b]	n/a	40
MIL-53(tdc)(Al)	1000	0.415	6.9[b]	83[b]	n/a	40
CAU-10-H	600	0.258	4.8[b]	25[b]	n/a	40
MIL-96(Al)	530	0.237	6.5[b]	8[b]	n/a	40
MIL-100(Al)	1890	0.824	16.3[b]	38[b]	n/a	40
NU-200	1260	0.52	11.7	80[f]	Y	41
P(Ph-4MVIm-Br)	158	0.12	8.12	18413[f]	N	42
Ph-4MVIm-Br	-	-	10.26	-	N	42
P(Ph-3MVIm-Br)	121	-	7.4	47335[f]	N	42
Ph-3MVIm-Br	-	-	10.52	-	N	42
P(Ph-2MVIm-Br)	110	-	6.71	-	N	42
Ph-2MVIm-Br	-	-	8.41	-	N	42
P(EVIm-Br)	-	-	10.51	-	N	42

[a.] The uptake was recorded at 298 K and 1 bar; [b.] the uptake was recorded at 293 K; [c.] the uptake was recorded at 273 K; [d.] the uptake was recorded to 0.92 bar; [e.] the uptake was recorded at 313 K; [f.] The ratio of SO_2:CO_2 mixture 10:90; [g.] The ratio of SO_2:CO_2 mixture 1:99; [h.] dynamic selectivity for the ratio of SO_2:CO_2 mixture 1:99; *MOF is unstable towards SO_2 sorption.

References

1. Lin, X.; Jia, J.; Zhao, X.; Thomas, K.M.; Blake, A.J.; Walker, G.S.; Champness, N.R.; Hubberstey, P.; Schröder, M. High H_2 Adsorption By Coordination Framework Materials. *Angew. Chem. Int. Ed.* **2006**, *45*, 7358-7364.
2. Rao, X.; Cai, J.; Yu, J.; He, Y.; Wu, C.; Zhou, W.; Yildirim, T.; Chen, B.; Qian, G. A Microporous Metal–Organic Framework With Both Open Metal And Lewis Basic Pyridyl Sites For High C_2H_2 And CH_4 Storage At Room Temperature. *Chem. Commun.* **2013**, *49*, 6719-6721.
3. Humby, J.; Benson, O.; Smith, G.; Argent, S.; da Silva, I.; Cheng, Y.; Rudić, S.; Manuel, P.; Frogley, M.; Cinque, G.; Saunders, L.; Vitórica-Yrezábal, I.; Whitehead, G.; Easun, T.; Lewis, W.; Blake, A.; Ramirez-Cuesta, A.; Yang, S.; Schröder, M. Host–Guest Selectivity In A Series Of Isoreticular Metal–Organic Frameworks: Observation Of Acetylene-To-Alkyne And Carbon Dioxide-To-Amide Interactions. *Chem. Sci.* **2019**, *10*, 1098-1106.
4. Sheldrick, G. SHELXT– Integrated Space-Group And Crystal-Structure Determination. *Acta Crystallogr. A,* **2015**, *71*, 3-8.
5. Sheldrick, G. Crystal Structure Refinement With SHELXL. *Acta Crystallogr. C Struct. Chem.* **2015**, *71*, 3-8.
6. Spek, A. PLATONSQUEEZE: A Tool For The Calculation Of The Disordered Solvent Contribution To The Calculated Structure Factors. *Acta Crystallogr. C Struct. Chem.* **2015**, *71*, 9-18.
7. Smith, G.; Eyley, J.; Han, X.; Zhang, X.; Li, J.; Jacques, N.; Godfrey, H.; Argent, S.; McCormick McPherson, L.; Teat, S.; Cheng, Y.; Frogley, M.; Cinque, G.; Day, S.; Tang, C.; Easun, T.; Rudić, S.; Ramirez-Cuesta, A.; Yang, S.; Schröder, M. Reversible Coordinative Binding And Separation Of Sulfur Dioxide In A Robust Metal–Organic Framework With Open Copper Sites. *Nat. Mater.* **2019**, *18*, 1358-1365.
8. Cui, X.; Yang, Q.; Yang, L.; Krishna, R.; Zhang, Z.; Bao, Z.; Wu, H.; Ren, Q.; Zhou, W.; Chen, B.; Xing, H. Ultrahigh And Selective SO_2 Uptake In Inorganic Anion-Pillared Hybrid Porous Materials. *Adv. Mater.* **2017**, *29*, 1606929.
9. Glomb, S.; Woschko, D.; Makhloufi, G.; Janiak, C. Metal–Organic Frameworks With Internal Urea-Functionalized Dicarboxylate Linkers For SO_2 And NH_3 Adsorption. *ACS Appl. Mater. Interfaces* **2017**, *9*, 37419-37434.
10. Yang, S.; Liu, L.; Sun, J.; Thomas, K.; Davies, A.; George, M.; Blake, A.; Hill, A.; Fitch, A.; Tang, C.; Schröder, M. Irreversible Network Transformation In A Dynamic Porous Host Catalyzed By Sulfur Dioxide. *J. Am. Chem. Soc.* **2013**, *135*, 4954-4957.
11. Tan, K.; Canepa, P.; Gong, Q.; Liu, J.; Johnson, D.; Dyevoich, A.; Thallapally, P.; Thonhauser, T.; Li, J.; Chabal, Y. Mechanism Of Preferential Adsorption Of SO_2 Into Two Microporous Paddle Wheel Frameworks $M(Bdc)(Ted)_{0.5}$. *Chem. Mater.* **2013**, *25*, 4653-4662.
12. Grant Glover, T.; Peterson, G.; Schindler, B.; Britt, D.; Yaghi, O. MOF-74 Building Unit Has A Direct Impact On Toxic Gas Adsorption. *Chem. Eng. Sci.* **2011**, *66*, 163-170.

13. Savage, M.; Cheng, Y.; Easun, T.; Eyley, J.; Argent, S.; Warren, M.; Lewis, W.; Murray, C.; Tang, C.; Frogley, M.; Cinque, G.; Sun, J.; Rudić, S.; Murden, R.; Benham, M.; Fitch, A.; Blake, A.; Ramirez-Cuesta, A.; Yang, S.; Schröder, M. Selective Adsorption Of Sulfur Dioxide In A Robust Metal–Organic Framework Material. *Adv. Mater.* **2016**, *28*, 8705-8711.
14. Lee, G.; Lee, J.; Vo, H.; Kim, S.; Lee, H.; Park, T. Amine-Functionalized Covalent Organic Framework For Efficient SO_2 Capture With High Reversibility. *Sci. Rep.* **2017**, *7*, 557.
15. Thallapally, P.; Motkuri, R.; Fernandez, C.; McGrail, B.; Behrooz, G. Prussian Blue Analogues For CO_2 And SO_2 Capture And Separation Applications. *Inorg.* **2010**, *49*, 4909-4915.
16. Fernandez, C.; Thallapally, P.; Motkuri, R.; Nune, S.; Sumrak, J.; Tian, J.; Liu, J. Gas-Induced Expansion And Contraction Of A Fluorinated Metal−Organic Framework. *Cryst. Growth Des.* **2010**, *10*, 1037-1039.
17. Carter, J.; Han, X.; Moreau, F.; da Silva, I.; Nevin, A.; Godfrey, H.; Tang, C.; Yang, S.; Schröder, M. Exceptional Adsorption And Binding Of Sulfur Dioxide In A Robust Zirconium-Based Metal–Organic Framework. *J. Am. Chem. Soc.* **2018**, *140*, 15564-15567.
18. Li, L.; da Silva, I.; Kolokolov, D.; Han, X.; Li, J.; Smith, G.; Cheng, Y.; Daemen, L.; Morris, C.; Godfrey, H.; Jacques, N.; Zhang, X.; Manuel, P.; Frogley, M.; Murray, C.; Ramirez-Cuesta, A.; Cinque, G.; Tang, C.; Stepanov, A.; Yang, S.; Schroder, M. Post-Synthetic Modulation Of The Charge Distribution In A Metal–Organic Framework For Optimal Binding Of Carbon Dioxide And Sulfur Dioxide. *Chem. Sci.* **2019**, *10*, 1472-1482.
19. Tchalala, M.; Bhatt, P.; Chappanda, K.; Tavares, S.; Adil, K.; Belmabkhout, Y.; Shkurenko, A.; Cadiau, A.; Heymans, N.; De Weireld, G.; Maurin, G.; Salama, K.; Eddaoudi, M. Fluorinated MOF Platform For Selective Removal And Sensing Of SO_2 From Flue Gas And Air. *Nat. Commun.* **2019**, *10*, 1328.
20. Martínez-Ahumada, E.; Díaz-Ramírez, M.; Lara-García, H.; Williams, D.; Martis, V.; Jancik, V.; Lima, E.; Ibarra, I. High And Reversible SO_2 Capture By A Chemically Stable Cr(Iii)-Based MOF. *J. Mater. Chem. A.* **2020**, *8*, 11515-11520.
21. Zárate, J.; Sánchez-González, E.; Williams, D.; González-Zamora, E.; Martis, V.; Martínez, A.; Balmaseda, J.; Maurin, G.; Ibarra, I. High And Energy-Efficient Reversible SO_2 Uptake By A Robust Sc(Iii)-Based MOF. *J. Mater. Chem. A.* **2019**, *7*, 15580-15584.
22. Guo, L.; Feng, X.; Gao, Z.; Krishna, R.; Luo, F. Robust 4D–5F Bimetal–Organic Framework For Efficient Removal Of Trace SO_2 From SO_2/CO_2 And $SO_2/CO_2/N_2$ Mixtures. *Inorg.* **2021**, *60*, 1310-1314.
23. Hungerford, J.; Bhattacharyya, S.; Tumuluri, U.; Nair, S.; Wu, Z.; Walton, K. DMOF-1 As A Representive MOF for SO2 Adsorption In Both Humid And Dry Conditions. *J. Phys. Chem. C* **2018**, 122, 23498-23500.
24. Tannert, N.; Sun, Y.; Hasturk, E.; Niebing, S.; Janiak, C. A Series of New Urea-MOFs Obtained *via* Post-Synthetic Modification of NH_2-MIL-101(Cr): SO_2, CO_2 and H_2O Sorption. *Z. Anorg. Allg. Chem.* **2021**, *647*, 1124–1130.

25. Briggs, L.; Newby, R.; Xue, H.; Morris, C.; Savage, M.; Krap, C.; Easun, T.; Frogley, M.; Cinque, G.; Murray, C.; Tang, C.; Sun, J.; Yang, S.; Schroder, M. Binding and Separation of CO_2, SO_2 and C_2H_2 in Homo- and Hetero-Metallic Metal-Organic Framework Materials. *J. Mater. Chem. A*, **2021**, *9*, 7190-7197.
26. Yin, M.; Xiong, X.; Feng, X.; Xu, W.; Krishna, R.; Luo, F. A Robust Cage-Based Metal－Organic Framework Showing Ultrahigh SO2 Uptake for Efficient Removal of Trace SO_2 from SO_2/CO_2 and $SO_2/CO_2/N_2$ Mixtures. *Inorg. Chem.* **2021**, *60*, 3447-3451.
27. Fan, Y.; Yin, M.; Krishna, R.; Feng, X.; Luo, F. Constructing A Robust Gigantic Drum-like Hydrophobic [$Co_{24}U_6$] Nanocage in A Metal-Organic Framework for High-performance SO_2 Removal in Humid Conditions. *J. Mater. Chem. A*, **2021**, *9*, 4075–4081.
28. Brandt, P.; Nuhnen, A.; Ozturk, S.; Kurt G.; Liang , J.; Janiak, C. Comparative Evaluation of Different MOF and Non-MOF Porous Materials for SO_2 Adsorption and Separation Showing the Importance of Small Pore Diameters for Low-Pressure Uptake. *Adv. Sustainable Syst.* **2021**, *5*, 2000285.
29. Rivera-Almazo, M.; Diaz-Ramirez, M.; Hernandez-Esparza, R.; Vargas, R.; Martinez, A.; Martis, V.; Saenz-Cavazos, P.; Williams, D.; Lima, E.; Ibarra, I.; Garza, J. Identification of the Preferential CO and SO_2 Adsorption Sites within NOTT-401. *Phys. Chem. Chem. Phys.,* **2021**, *23*, 1454-1463.
30. Fan, Y.; Zhang, H.; Yin, M.; Krishna, R.; Feng, X.; Wang, L.; Luo, M.; Luo, F. High Adsorption Capacity and Selectivity of SO_2 over CO_2 in A Metal-Organic Framework. *Inorg. Chem.* **2021**, *60*, 4–8.
31. Gorla, S.; Diaz-Ramirez, M.; Abeynayake, N.; Kaphan, D.; Williams, D.; Martis, V.; Lara-Garcia, H.; Donnadieu, B.; Lopez, N.; Ibarra, I.; Montiel-Palma, V. Functionalized NU-1000 with An Iridium Organometallic Fragment: SO_2 Capture Enhancement. *ACS Appl. Mater. Interfaces* **2020**, *12*, 41758−41764.
32. Zarate, J.; Dominguez-Ojeda, E.; Sanchez-Gonzalez, E.; Martinez-Ahumada, E.; Lopez-Cervantes, V.; Williams, D.; Martis, V.; Ibarra, I.; Alejandre, J. Reversible and Efficient SO_2 Capture by A Chemically Stable MOF CAU-10: Experiments and Simulations. *Dalton Trans.*, **2020**, *49,* 9203–9207.
33. Zhang, Y.; Chen, Z.; Liu, X.; Dong, Z.; Zhang, P.; Wang, J.; Deng, Q.; Zeng, Z.; Zhang, S.; Deng, S. Efficient SO_2 Removal Using a Microporous Metal–Organic Framework with Molecular Sieving Effect. *Ind. Eng. Chem. Res.* **2020**, *59*, 874−882.
34. Dominguez-Gonzalez, R.; Rojas-Leon, I.; Martinez-Ahumada, E.; Martinez-Otero, D.; Lara-Garcia, H.; Balmaseda-Era, J. Ibarra, I.; Percastegui, E.; Jancik, V. UNAM-1: A Robust Cu^I and Cu^{II} Containing 3D-Hydrogen-Bonded Framework with Permanent Porosity and Reversible SO_2 Sorption. *J. Mater. Chem. A*, **2019**, *7*, 26812–26817.
35. Zhang, Y.; Zhang, P.; Yu, W.; Zhang, J.; Huang, J.; Wang, J.; Xu, M.; Deng, Q.; Zeng, Z.; Deng, S. Highly Selective and Reversible Sulfur Dioxide Adsorption on a Microporous Metal–Organic Framework *via* Polar Sites. *ACS Appl. Mater. Interfaces* **2019**, *11*, 10680−10688.

36. Zhang, Z.; Yang, B.; Wu, Y.; Zhang, W.; Ma, H. Post Modification of Oxo-clusters in Robust Zirconium-Based Metal Organic Framework for Durable SO_2 Capture from Flue Gas. *Separation and Purification Technology* **2021**, *276,* 119349.
37. Ponce, J.; Diaz-Ramirez, M.; Gorla, S.; Navarathna, C.; Sanchez-Lecuona, G.; Donnadieu, B.; Ibarra, I.; Montiel-Palma, V. SO_2 Capture Enhancement in NU-1000 by the Incorporation of A Ruthenium Gallate Organometallic Complex. *CrystEngComm*, **2021**, *23*, 7479–7484.
38. Sun, Y.; Liang, J.; Brandt, P.; Spieb, A.; Ozturk, S.; Janiak, C. Cucurbit[6]uril@MIL-101-Cl: Loading Polar Porous Cages in Mesoporous Stable Host for Enhanced SO_2 Adsorption at Low Pressures. *Nanoscale*, **2021**, *13*, 15952–15962.
39. Lopez-Olvera, A.; Zarate, J.; Martinez-Ahumada, E.; Fan, D.; Diaz-Ramiirez, M.; Saenz-Cavazos, P.; Martis, V.; Williams, D.; Sanchez-Gonzalez, E.; Maurin, G.; Ibarra, I. SO_2 Capture by Two Aluminum-Based MOFs: Rigid-like MIL-53(Al)- TDC versus Breathing MIL-53(Al)-BDC. *ACS Appl. Mater. Interfaces* **2021**, *13*, 39363−39370.
40. Brandt, P.; Xing, S.; Liang, J.; Kurt, G.; Nuhnen, A.; Weingart, O.; Janiak, C. Zirconium and Aluminium MOFs for Low-Pressure SO_2 Adsorption and Potential Separation: Elucidating the Effect of Small Pores and NH_2 Groups. *ACS Appl. Mater. Interfaces* **2021**, *13*, 29137–29149.
41. Gong, W.; Xie, Y.; Pham, T.; Shetty, S.; Son, F.; Idrees, K.; Chen, Z.; Xie, H.; Liu, Y.; Snurr, R.; Chen, B.; Alameddine, B.; Cui, Y.; Farha, O. Creating Optimal Pockets in a Clathrochelate-based Metal-organic Framework for Gas Adsorption and Separation: Experimental and Computational Studies. *J. Am. Chem. Soc.* **2022**, *144*, 3737-3745.
42. Suo, X.; Yu, Y.; Qian, S.; Zhou, L.; Cui, X.; Xing, H. Tailoring the Pore Size and Chemistry of Ionic Ultramicroporous Polymers for Trace Sulfur Dioxide Capture with High Capacity and Selectivity. *Angew. Chem. Int. Ed.* **2021**, *60*, 6986-6991.
